# Endogenous Aquaporin‑0 Lipid Binding in Ocular Lens Tissue via Native Mass Spectrometry

**DOI:** 10.1021/jasms.4c00500

**Published:** 2025-06-24

**Authors:** Carla V.T. O’Neale, Sophie R. Harvey, Sergei Chetyrkin, Vicki H. Wysocki, Kevin L. Schey

**Affiliations:** † Department of Biochemistry, 5718 Vanderbilt University, Nashville, Tennessee 37205, United States; ‡ Department of Chemistry and Biochemistry and Native Mass Spectrometry Guided Structural Biology Center, 2647 The Ohio State University, Columbus, Ohio 43210, United States; § Mass Spectrometry Research Center, Vanderbilt University, Nashville, Tennessee 37240, United States; ∥ School of Chemistry and Biochemistry, Georgia Tech, Atlanta, Georgia 30332, United States

## Abstract

The ocular lens microcirculation system (MCS) is required to maintain transparency; however, how this system is established and maintained as a function of age is not well understood. Through its role in cell adhesion and water permeability, Aquaporin-0 (AQP0) is an important protein in the generation and regulation of the MCS. AQP0 permeability studies have shown that the lipid composition surrounding AQP0 has a direct effect on its function; nevertheless, interactions of native lens lipids with AQP0 have yet to be elucidated. In this study, we used native mass spectrometry (nMS) analysis of ocular lens membrane preparations to identify endogenous lipids bound to AQP0 to inform our understanding of how AQP0-lipid interactions regulate AQP0 function in the lens. We found that a variety of endogenous lens lipids (phosphatidylcholines (PCs) and sphingomyelins (SMs)) differentially bind AQP0 in a regionally dependent manner (cortex vs nucleus). Furthermore, spike-in experiments using native lens lipid extracts allowed us to uncover new AQP0-lipid assemblies not detected in the crude AQP0 experiments, including AQP0-ether-linked PC and AQP0-SM interactions.

## Graphical abstract

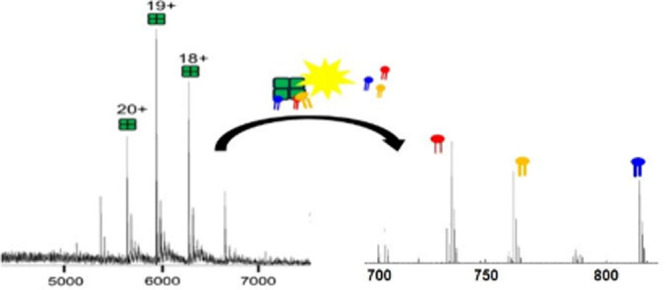

## Introduction

The ocular lens is an avascular organ that refracts light onto the retina and is necessary for high visual acuity. Lens transparency, critical for clear vision, is maintained in the lens through a unique cellular architecture of fiber cells, proteins, and membrane lipids. Lipids play a vital role in the lens by not only forming membrane barriers to molecular diffusion, but by creating local environments that can affect the function of lens membrane proteins, such as aquaporins.^1^ Aquaporins belong to the major intrinsic protein family of water channels and are composed of six transmembrane α-helical domains and N- and C- termini in the cytoplasm.^2^ These differentially distributed water channels play a fundamental role in conducting water through the lens and in maintaining lens homeostasis through what is known as the lens microcirculation system (MCS).^3^ The most abundant membrane protein in the lens, aquaporin-0 (AQP0), functions as both a water channel^4^ and a cell-adhesion protein^5^ and plays a critical role in the lens microcirculation system.^6^

AQP0 function is regulated by pH,^7^ protein interactions, such as with calmodulin^8–11^ and filensin,^12,13^ C-terminal cleavage^14^ and membrane lipids.^15^ Studies of AQP0-lipid interactions are limited but include electron crystallographic structures of lens AQP0 in DMPC^16^ and in *E. coli* polar lipids,^17^ and recently in sphingomyelin/cholesterol bilayers.^18^ Importantly, Tong et al.,^15^ showed that when AQP0 was reconstituted into proteoliposomes composed of different lipid mixtures, its water permeability was dependent on the lipid bilayer composition. Specifically, AQP0 in bilayers composed of lipids characteristic of lens fiber cell membranes, i.e. sphingomyelin and cholesterol, displayed a lower unit water permeability compared to bilayers containing phosphatidylcholine and phosphatidylglycerol.^15^ The study suggested that AQP0 function within the lens is spatially dependent, specifically that AQP0 water permeability is higher in the cortical region compared to the nuclear region which is high in sphingomyelin and cholesterol.^15^

Given the importance of lipids in regulating AQP0s function, we sought to investigate AQP0-lipid interactions, for the first time, with endogenous lipids. To expand our understanding of AQP0-lipid interactions, we employed native mass spectrometry (nMS) to identify endogenous lens lipids that directly interact with AQP0. Native MS complements traditional structural biology techniques by analyzing intact proteins in complex with their ligands.^19,20^ In this study, AQP0 was isolated from bovine lens tissue and analyzed via nMS in crude membrane preparations to preserve endogenous noncovalent lipid interactions. Additionally, native lens lipids were also spiked into purified AQP0 to uncover AQP0-lipid interactions that were undetected in the crude sample preparations. Several different native lipids, identified by LC-MS/MS, were found to interact with AQP0 in a lens region-dependent manner.

## Methods

### Bovine Lens Dissection and Tissue Washes

Bovine lenses from Pel-Freez Biologicals (Rogers, AK; cat no. 57114-2) stored at −80 °C, 12–30 months of age, were decapsulated and the lens cortex (∼2.5 mm thickness) was manually dissected from the nucleus and prepared independently. Using a glass dounce homogenizer, each sample was manually homogenized in cold homogenizing buffer (25 mM Tris-HCl, 1 mM PMSF (phenylmethylsulfonyl fluoride), 5 mM EDTA, 150 mM NaCl, pH 7.5) and centrifuged at 100,000*g* for 20 min at 4 °C, the supernatants were discarded, and the pellets were retained. Each pellet was resuspended a second time in homogenizing buffer, vortexed briefly, and centrifuged as described. The supernatants were discarded and the pellets retained. As described below, the pellets were further washed depending on the experiment.

Crude cortex and crude nucleus sample preparation for native MS was carried out as follows. Cortex and nucleus pellets were independently resuspended in 4 M urea in Tris buffer (25 mM Tris-HCl, 5 mM EDTA, 150 mM NaCl, pH 7.5), vortexed briefly, and centrifuged as described. The supernatants were discarded, and this was repeated three additional times for a total of four 4 M urea washes. The pellets were then washed one time with 25 mM Tris-HCl, pH 7.5 to remove residual urea followed by centrifugation as described and the supernatants discarded. These washed crude cortex and nucleus pellets were flash frozen on dry ice and stored at −80 °C until use for native MS or for LC-MS/MS analysis.

### Purification of AQP0 via Anion-Exchange Chromatography

Nucleus pellets (prepared as described above) used for AQP0 purification were resuspended in 4 M urea in Tris buffer (25 mM Tris-HCl, 5 mM EDTA, 150 mM NaCl, pH 7.5), vortexed briefly and centrifuged as described. This was repeated using 8 M urea in Tris buffer. The pellet was then washed with water, cold 100 mM NaOH in Tris buffer and then two times with water, with each of these washes followed by centrifugation as described and the supernatant discarded. These washed nucleus pellets were flash frozen on dry ice and stored at −80 °C until purification as described below.

The washed nucleus pellet was thawed and AQP0 was purified as previously described.^21^ The pellet was resuspended in 2% n-octyl-ß-d-glucopyranoside (OG) (RPI; cat no. N02007) in Tris buffer, vortexed, incubated on ice for a minimum of 30 min and centrifuged at 100,000*g* for 10 min at 4 °C. The supernatant was loaded onto a self-packed anion exchange column (Source 15Q; GE Healthcare 17–0947–20) and AQP0 was purified via the following step gradient at 0.70 mL/min: 0–10 min (100% buffer A: 25 mM Tris-HCl, 1% OG, pH 7.5), 10–20 min (92.5% buffer A and 7.5% buffer B: 2 M NaCl, 25 mM Tris-HCl, 1% OG, pH 7.5) and 20–30 min (85% buffer A and 15% buffer B). Proteins were detected by 220 nm absorbance. Purified AQP0 was concentrated using a 4 mL-50 kDa MWCO Amicon Ultra centrifugal filter (Millipore cat no. UFC805024) and protein concentration was measured via bicinchoninic acid (BCA) assay or A280 using a NanoDrop 2000.

### Sample Preparation for Native MS Analysis

Crude cortex and crude nucleus pellets were individually thawed and prepared. Each pellet was solubilized in 1% OG in 25 mM Tris-HCl, pH 7.5 and incubated overnight at 4 °C with end-overend rotation. Samples were centrifuged at 100,000*g* for 10 min at 4 °C and the supernatant was collected, loaded onto a 500 μL- 100 kDa MWCO Amicon Ultra centrifugal filter (Millipore cat no. UFC5100), and concentrated and buffer exchanged nine times into native MS buffer (200 mM ammonium acetate (AmAc), 2x critical micelle concentration of tetraethylene glycol monooctyl ether (C_8_E_4_)). It should be noted that incomplete removal of OG may have resulted in mixed micelles in this analysis. The final protein concentration was measured via bicinchoninic acid (BCA) assay or A280 using a NanoDrop 2000.

Concentrated and purified AQP0 in 1% OG, 25 mM Tris-HCl, pH 7.5 was diluted with 200 mM AmAc, 2x CMC C8E4 and loaded onto a 500 μL-100 kDa MWCO Amicon Ultra centrifugal filter (Millipore cat no. UFC5100), reconcentrated and buffer exchanged three times into native MS buffer. It should be noted that incomplete removal of OG may have resulted in mixed micelles in this analysis. Samples were diluted with native MS buffer and directly infused into a Q Exactive Ultra High Mass Range (UHMR) mass spectrometer.

### Preparation of Exogenous Lipids for Native MS Spike-in Experiments

One milligram of 1,2-dipalmitoyl-*sn*-glycero-3-phosphocholine (DPPC; Avanti) powder in a glass vial was dissolved in chloroform and evaporated. The resulting lipid film was reconstituted in native MS buffer (200 mM ammonium acetate, 2x CMC C8E4) to a concentration of 510 μM and sonicated in a water bath sonicator for 10 min followed by sonication with a probe sonicator, 5–10x for 10 s each time, and then diluted with native MS buffer to a stock concentration of 100 μM. Purified AQP0 in 1% OG, 25 mM Tri-s-HCl, pH 7.5 (prepared as described above) was buffer exchanged 3x with native MS buffer and then gently combined at room temperature with DPPC for a final concentration of 3–4 μM of AQP0 and 8 μM of DPPC and immediately analyzed on the QE UHMR as described in Native Mass Spectrometry Analysis.

### Preparation of Endogenous Lens Lipids for Native MS Spike-in Experiments

A washed cortex pellet from one bovine lens (as described above) was thawed and reconstituted into water and transferred to a glass vial. Lipids were then extracted using the method of Bligh and Dyer^22^ and the resulting extract is referred to as endogenous lens lipid extract. Native lens lipid extracts were evaporated under a stream of nitrogen gas and frozen at −20 °C or used immediately for experiments. The lipid extract was reconstituted in 2 mL of native MS buffer and sonicated for 5–10 min in a water bath sonicator, followed by sonication with a probe sonicator, 10x for 10 s each time. For the AQP0 lens lipid spike-in sample, 55 μL of purified AQP0 (11 μM) was combined with 450 μL of the endogenous lens lipid extract stock. For the AQP0 sample (control), 25 μL of purified AQP0 was combined with 450 μL of native MS buffer. Samples were incubated overnight at 4 °C with end-overend rotation. Samples were then centrifuged for 5 min at 20,000*g* and each supernatant was loaded onto a 500 μL-100 kDa MWCO Amicon Ultra centrifugal filter (Millipore cat no. UFC5100) and buffer exchanged/reconcentrated one time with native MS buffer. Final concentration was measured via A280 using a NanoDrop 2000. Samples were analyzed via nMS as described in Native Mass Spectrometry Analysis or analyzed via LC-MS/MS as described in LC-MS/MS Lipid Extraction and Data Acquisition.

### Native Mass Spectrometry Analysis

Native mass spectrometry experiments were performed on a Thermo Fisher Scientific Q Exactive UHMR mass spectrometer. Samples in native MS buffer were introduced into the mass spectrometer by static nanospray ionization using in-house pulled borosilicate capillaries (Sutter Instrument, cat no. BF100–78–10). A platinum wire was inserted into the sample loaded capillary and an electrospray voltage of 1.2–1.3 kV was applied. Capillary temperature was set to 225 °C, and the trap gas flow rate was 5. The resolution was set to 25,000 or 50,000 for all experiments. With in-source trapping mode, a desolvation voltage of −60 to −70 was used. For MS/MS experiments, HCD fragmentation was performed at 200 V. The RF on the HCD cell and C-trap were tuned for low mass transfer, specifically the injection flatapole amplitude was 700 V, bent flatapole amplitude was 940 V, transfer multipole and HCD-cell RF amplitude was 250 V, and the C-Trap RF amplitude was 2300 V. Trap gas flow rate was 2.

### LC-MS/MS Lipid Extraction and Data Acquisition

Crude AQP0 from the cortex in native MS buffer was spiked with 10 μL equiSPLASH-lipidomics internal standard mix (Avanti Polar Lipids/CRODA), transferred to a glass tube and extracted using the method of Bligh and Dyer.^22^ The lipid extract was evaporated under a gentle stream of nitrogen gas and resuspended in 100 μL of HPLC-grade methanol and chloroform (9:1).

Discovery lipidomics analysis was performed at Vanderbilt’s Mass Spectrometry Research Center (MSRC) MS Core facility using a Vanquish ultrahigh performance liquid chromatography (UHPLC) system interfaced to a Q Exactive HF quadrupole/orbitrap mass spectrometer (Thermo Fisher Scientific) operating in data-dependent acquisition mode. Sample (5 μL) was injected and analyzed in both positive and negative ESI modes.

Chromatographic separation was performed with a reverse-phase Acquity BEH C18 column (1.7 um, 2.1 × 150 mm, Waters, Milford, MA) at a flow rate of 250 μL/min. Mobile phases were made up of 10 mM ammonium formate and 0.1% formic acid in (A) H_2_O/CH_3_CN (40:60) and in (B) CH_3_CN/iPrOH (10:90). Gradient conditions were as follows: 0–1 min, B = 20%; 1–8 min, B = 20– 100%; 8–10 min, B = 100%; 10–10.5 min, B = 100–20%; 10.5–15 min, B = 20%. Mass spectra were acquired over a precursor ion range of *m*/*z* 200 to 1,600 at a resolving power of 60,000 using the following HESI-II source parameters: spray voltage 4 kV; capillary temperature 250 °C; S-lens RF level 60 V; nitrogen sheath gas 40; nitrogen auxiliary gas 10; auxiliary gas temperature 350 °C. MS/MS spectra were acquired for the top-seven most abundant precursor ions with an MS/MS automatic gain control (AGC) target of 1e5, a maximum MS/MS injection time of 100 ms, and a normalized collision energy of 15, 30, 40.

High resolution mass spectrometry data was processed with MS-DIAL version 4.90 in lipidomics mode.^23^ MS1 and MS2 tolerances were set to 0.01 and 0.025 Da, respectively. Minimum peak height was set to 30,000 to decrease the number of false positive hits. Peaks were aligned with retention time tolerance of 0.1 min and mass tolerance of 0.015 Da. A default lipid library was used (Msp20210527163602_converted.lbm2), solvent type was set to HCOONH_4_ to match the solvent used for separation, and the identification score cut off was set to 80%. All lipid classes were made available for the search. After lipid identification was completed, MS-DIAL results were exported into Excel and filtered using maximum allowed relative standard deviation (RSD) for QC samples set to 25% and minimum allowed ratio of sample to blank of 10.

MetaboAnalyst version 5.0 ^24^ was used to perform statistical calculations on a combined list of all annotated lipids (both positive and negative ionization modes) which passed quality control filters described above. Lipids with a fold change >2 and false discovery rate (FDR) < 0.05 were considered significantly different.

Tandem mass spectra for all identified lipids are available in Supplementary Figures 1–21.

## Results

Native MS was previously employed to examine different proteoforms of intact purified AQP0 by noncovalent complex fragmentation through collision-induced dissociation (CID) and surface-induced dissociation (SID).^21^ Various AQP0 proteoforms were detected including phosphorylated, oleated and truncated forms.^21^ The study revealed novel AQP0 structural information, such as doubly phosphorylated AQP0 tetramers were composed of two singly phosphorylated monomers, and various truncated AQP0 monomers were present within a single tetramer.^21^ This previous study utilized purified AQP0, which, likely due to the purification process, was largely stripped of noncovalent lipid interactions. An additional study by Hale and Cooper,^25^ using native ambient mass spectrometry, remarkably reported intact AQP0 directly from sheep lens tissue sections; however, no AQP0-lipid interactions were reported.

In the current study, AQP0 was isolated from bovine lens cortex and nucleus regions and enriched through a series of washes that removed most soluble proteins. To preserve noncovalent AQP0 lipid interactions, AQP0 was analyzed in a complex mixture, referred to as crude AQP0, in this study. In addition, purified AQP0 was incubated with an individual exogenous lipid as well as a lens lipid extract to verify observations from crude AQP0 preparations.

### Crude AQP0 from the Lens Cortex

Figure 1a shows a mass spectrum of tetrameric AQP0 from the crude bovine lens cortex preparation. The most abundant species is full-length AQP0 with an average mass of 112,898 ± 0.6 Da, with lower abundance species also detected, including singly- and doubly- phosphorylated (P) AQP0 tetramers. The inset shows a zoomed-in view of the 19 + charge state, indicating several peaks (highlighted in yellow) representing putatively lipid bound AQP0. Three specific adduct peaks were annotated, + 729, + 810 and +893 and we suspect that these peaks represent AQP0 bound to different lipids as explained in the Discussion and/or one main lipid (*m*/*z* 734; DPPC) bound to the unphosphorylated, singly and doubly phosphorylated forms of AQP0 The signals in the highlighted region were isolated for tandem MS and, higher energy collision induced dissociation (HCD) fragmented AQP0 into predominantly monomers (extended mass spectrum and tandem mass spectrum available in Supplementary Figures 22 and 23, respectively) and released several noncovalently bound lipids as shown in Figure 1b. The Table inset Figure 1b lists the different lipid *m*/*z* detected in the tandem mass spectrum and their identities determined by LC-MS/MS analysis of this crude bovine cortex sample. Tandem mass spectra of all identified lipids in this study are shown in Supplementary Figures 1–21.

**Figure 1:**
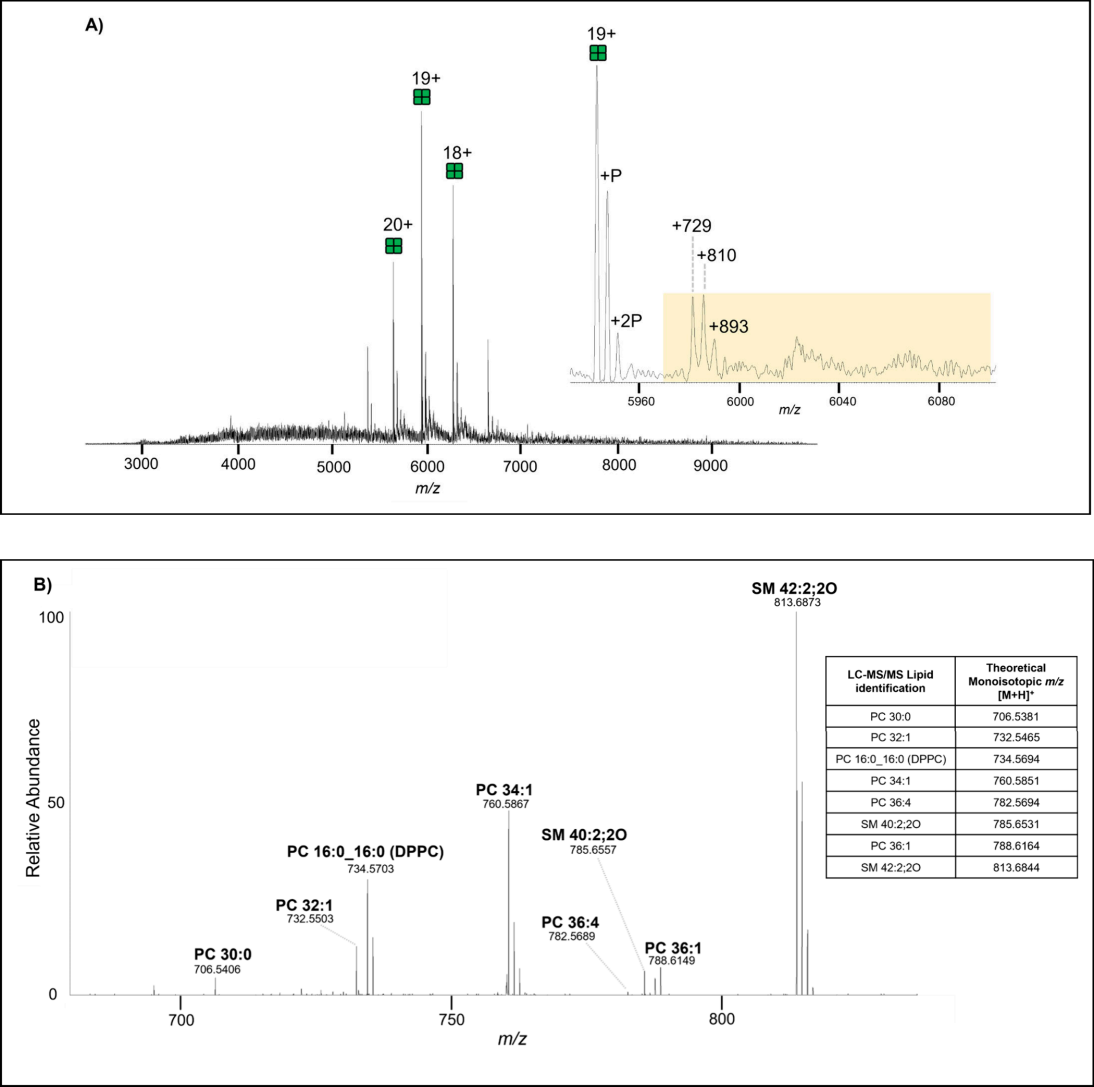
(A) Mass spectrum of tetrameric AQP0 from a crude bovine cortex acquired on a Q Exactive UHMR spectrometer. Phosphorylated (P) AQP0 is labeled as well as putative lipid adducts (measured centroid to centroid from 19+ unmodified tetramer). (Inset) Zoom-in of the 19+ charge state, highlighting the isolation window (5,970–6,100 *m*/*z*) for MS/MS analysis. Extended mass spectrum available in Supplementary Figure 22. (B) Tandem spectrum reveals the *m*/*z* values of lipids released from crude cortex AQP0 after fragmentation at 200 V of the signals in the isolation window highlighted in (A). Extended tandem mass spectrum is available in Supplementary Figure 23. Data were acquired at 25,000 resolution. Inset) LC-MS/MS identification of the lipids detected via nMS of an AQP0 crude bovine lens sample (Supplementary Figures 1–21). Calculated mass error reported in Supplementary Table 1.

### Crude AQP0 from the Lens Nucleus

Figure 2a shows a mass spectrum of crude tetrameric AQP0 from the bovine lens nucleus. Like the cortex, the major signals observed represent full-length AQP0 with an average mass of 112,898 ± 1 Da with lower abundance peaks representing singly- and doubly- phosphorylated AQP0 tetramers. The inset, a zoomed-in view of the 19 + charge state, shows several peaks (highlighted in yellow), representing putatively lipid bound AQP0, three of which were annotated, + 734, + 814 and +891. The signals in the highlighted region were isolated for tandem MS, and HCD fragmented AQP0 into mainly monomers (extended mass spectrum and tandem mass spectrum available in Supplementary Figures 24 and 25, respectively) and released several noncovalently bound lipids as shown in Figure 2b. The Table inset in Figure 2b lists the lipid masses detected in the native mass spectrum and their identities determined by LC-MS/MS. Tandem mass spectra of all identified lipids in this study are shown in Supplementary Figures 1–21.

**Figure 2:**
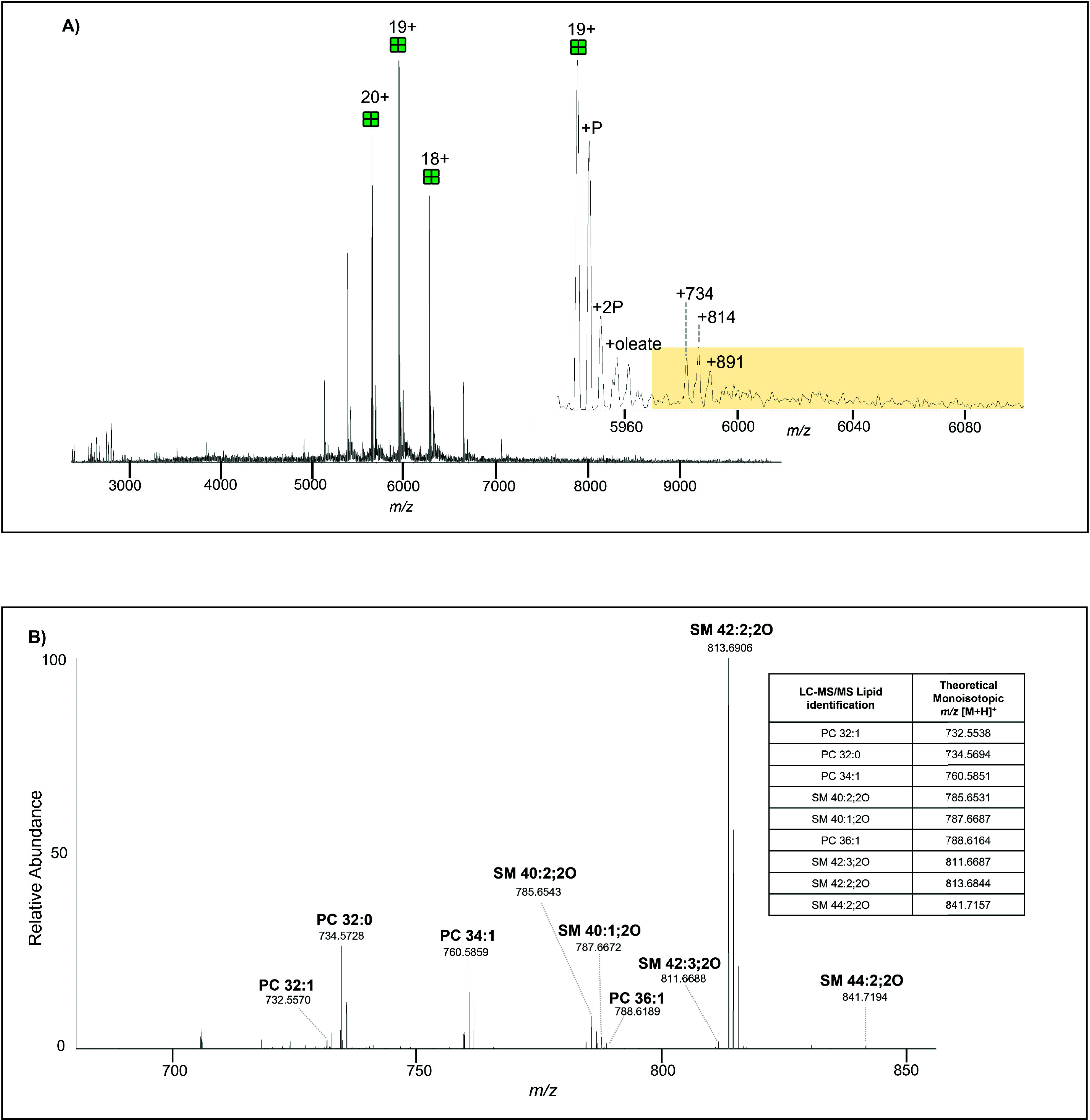
(A) Mass spectrum of tetrameric AQP0 from a crude bovine nucleus acquired on a Q Exactive UHMR spectrometer. Phosphorylated (P) AQP0 is labeled as well as putative lipid adducts (measured centroid to centroid from 19+ unmodified tetramer). (Inset) Zoom-in of the 19+ charge state, highlighting the isolation window (5,970–6,100 *m*/*z*) for MS/MS analysis. Extended mass spectrum available in Supplementary Figure 24. (B) Tandem mass spectrum reveals the *m*/*z* values of lipids released from crude nucleus AQP0 after fragmentation at 200 V of the signals in the isolation window highlighted in (A). Extended tandem mass spectrum is available in Supplementary Figure 25. MS data acquired at 25,000 and tandem MS acquired at 50,000 resolution. Inset) Lipids from an AQP0 crude bovine lens sample detected by LC-MS/MS to confirm the identity of the lipids detected via nMS. All lipid identifications were confirmed by MS/MS (tandem mass spectra are available for each identified lipid in Supplementary Figures 1–21). Calculated mass error reported in Supplementary Table 2.

### Purified AQP0 and DPPC Spike-in

To further confirm the identity of one of the AQP0 bound lipids (theoretical *m*/*z* 734.5694) via native MS, we performed spike-in experiments with purified AQP0 and DPPC (neutral MW:733 Da). Figure 3a shows a mass spectrum of purified AQP0 without DPPC and the inset highlights the area where the expected mass of AQP0 plus one DPPC molecule would appear. Figure 3b shows a mass spectrum of purified AQP0 with DPPC spiked-in showing a new peak (highlighted) corresponding to tetrameric 19+ AQP0 plus one DPPC molecule. The tandem MS of purified AQP0 without DPPC and with DPPC is reported in Supplementary Figures 27 and 28, respectively. The tandem MS of purified AQP0 with DPPC reveals a strong fragment ion (*m*/*z* 734.5721), consistent with DPPC, in the low mass range.

**Figure 3:**
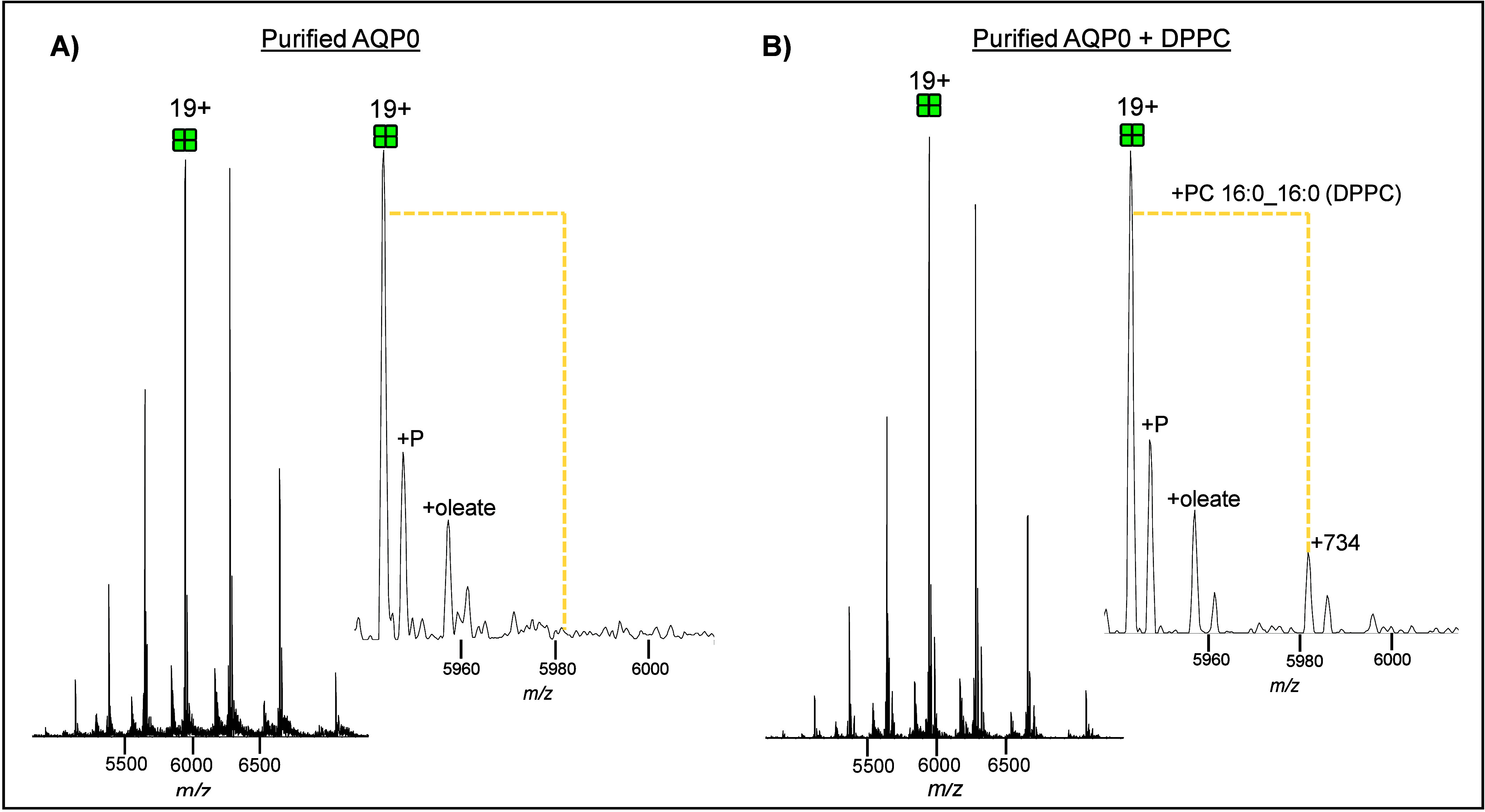
Mass spectra of purified AQP0 without (A) and with (B) exogenous DPPC spiked in. Insets show zoomed-in view of the 19+ charge state, highlighting (dashed yellow line) *m*/*z* region that would be expected for a DPPC adduct. Phosphorylated (P) AQP0 is labeled. Supplementary Figures 27 and 28 contain the MS/MS spectra for (A) and (B), respectively.

### Purified AQP0 and Endogenous Lens Lipid Spike-in

To reveal AQP0-lipid interactions that may have gone undetected in the crude AQP0 studies due to low abundance, lens lipids were extracted separately from the bovine lens cortex and nucleus, incubated overnight with purified AQP0, and then analyzed via native MS. Figures 4a and 5a display zoomed-in mass spectra of purified tetrameric AQP0. Regions that indicate possible AQP0-lipid complexes are highlighted in yellow. Figures 4b and 5b show zoomed-in mass spectra of purified tetrameric AQP0 incubated with cortical and nucleus lens lipid extracts, respectively. Regions in yellow highlight new and/or more intense peaks that appeared after overnight incubation in the lens lipid extract compared to purified AQP0 alone. Tandem mass spectra for AQP0 incubated in both lipid extracts show that several of the same lipids are found in both extracts and bind AQP0 (PC 16:0_16:0, PC 30:0, PC 32:1, PC 34:1, PC 36:1, PC 33:0, SM 34:1;2O, SM 40:2;2O, and SM 42:2;2O). However, there is one lipid (PC 36:2) that only binds AQP0 in the cortical lens lipid extract and various lipids that only bind to AQP0 (PC O-30:0, PC O-32:1, PC O-32:0, PC O-34:2, PC O-34:1, SM 40:1;2O, SM 41:1;2O, SM 42:3;2O, SM 44:2;2O, SM 44:3;2O) from the nucleus lens lipid extract including SMs and ether-linked PCs.

**Figure 4:**
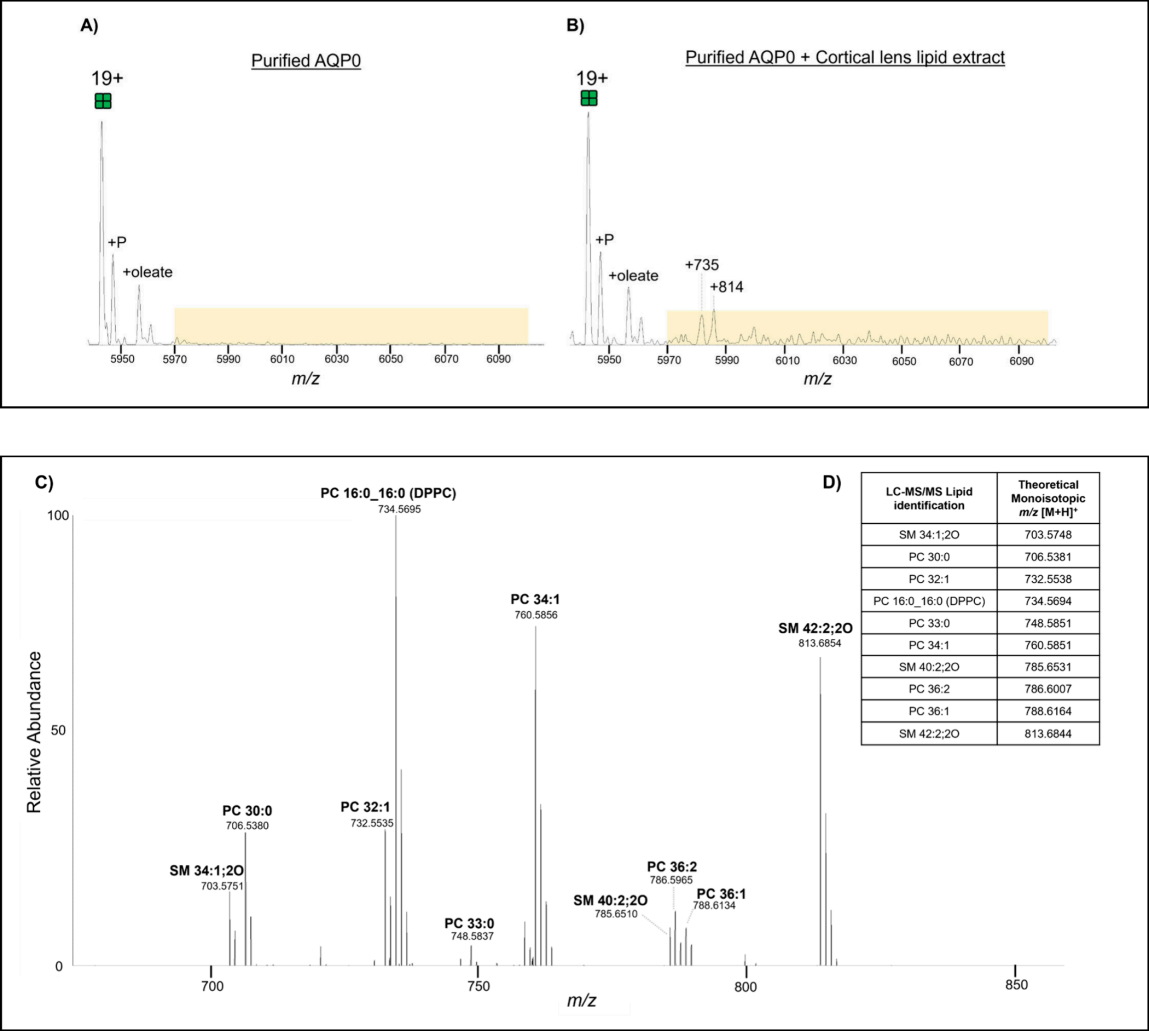
Zoomed-in view of mass spectra of 19+ charge state tetrameric purified AQP0 before (A) and after (B) incubation with a cortical lens lipid extract. Phosphorylated (P) AQP0 is labeled as well as putative lipid adducts (measured centroid to centroid from 19+ unmodified tetramer). Yellow highlighted regions indicate isolation window (5,970–6,100 *m*/*z*) selected for MS/MS. Data were acquired at 25,000 resolution. Extended mass spectra for (A) and (B) are available in Supplementary Figures 26 and 29, respectively. (C) The zoomed-in tandem spectrum reveals the *m*/*z* values of lipids released from purified AQP0 incubated with a cortical lens lipid extract after fragmentation at 200 V of the isolation window highlighted in (B). Extended tandem mass spectrum is available in Supplementary Figure 30. (D) Table listing the lipids from an AQP0 crude bovine lens sample detected by LC-MS/MS to confirm the identity of the lipids detected via nMS. All lipid identifications were confirmed by MS/MS (tandem mass spectra are available for each identified lipid in Supplementary Figures 1–21). Data acquired at 25,000 resolution. Calculated mass error reported in Supplementary Table 3.

**Figure 5:**
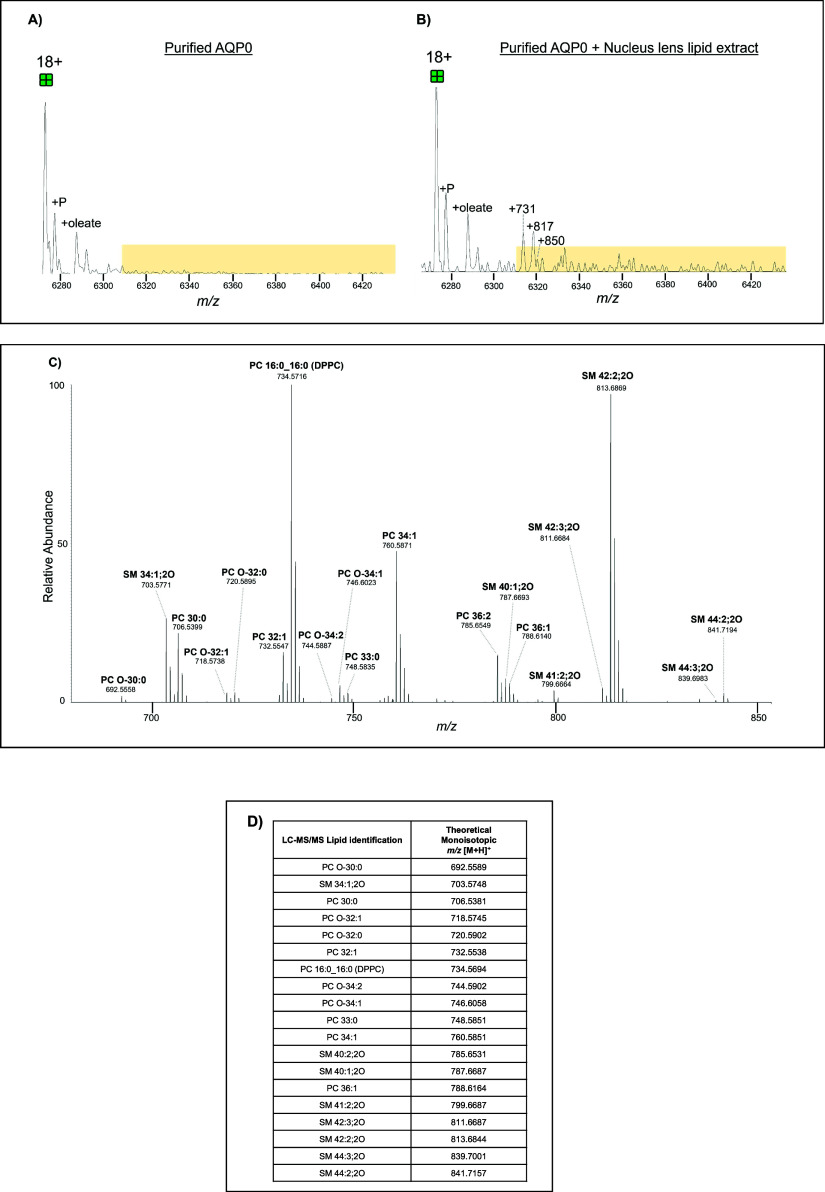
Zoomed-in view of mass spectra of 18+ charge state tetrameric purified AQP0 before (A) and after (B) incubation with nucleus lens lipid extract. Phosphorylated (P) AQP0 is labeled as well as putative lipid adducts (measured centroid to centroid from 18+ unmodified tetramer). Yellow highlighted regions indicate isolation window (6,310–6,440 *m*/*z*) selected for MS/MS. Extended mass spectra for (A) and (B) are available in Supplementary Figures 26 and 31, respectively. (C) The zoomed-in tandem spectrum reveals the *m*/*z* values of lipids released from purified AQP0 incubated with a nucleus lens lipid extract after fragmentation at 200 V of the isolation window highlighted in Figure 5B. Data were acquired at 25,000 resolution. Extended tandem mass spectrum is available in Supplementary Figure 32. (D) Table listing the lipids from an AQP0 crude bovine lens sample detected by LC-MS/MS to confirm the identity of the lipids detected via nMS. All lipid identifications were confirmed by MS/MS (tandem mass spectra are available for each identified lipid in Supplementary Figures 1–21). Calculated mass error reported in Supplementary Table 4.

### LC-MS/MS Lipid Identification

To identify the AQP0 bound lipids detected via nMS a separate lipidomics analysis was performed. Lipids were extracted from crude AQP0 and analyzed via LC-MS/MS to identify each lipid. Many lipid classes were identified including phosphatidylcholines (PCs), sphingomyelins (SMs), diacylglycerols (DGs) and phosphatidylelthanolamines (PEs). However, only PCs and SMs were identified in the native MS fragmentation experiments. The tandem mass spectra (derived from the lipidomics analysis) for each lipid identified in the native MS experiments is reported in Supplementary Figures 1–21.

## Discussion

In this study, we sought to use the unique features of native MS to detect noncovalently bound lipids to endogenous bovine lens AQP0 since AQP-lipid interactions are known to affect AQP permeability. In our experiments, several putative noncovalent lipids bound to AQP0 tetramers from bovine lens tissue were transferred into the gas-phase, fragmented from the tetramer, and identified by accurate mass measurement and LC-MS/MS analysis. Remarkably, these lipids remained noncovalently bound to AQP0 through the sample preparation process suggesting a strong affinity to AQP0. All lipids detected via native MS were structurally characterized by LC-MS/MS.

Interestingly, the human lens displays a regional difference in the distribution of lipids where, in the cortex, there is a lower amount of sphingomyelin compared to the nucleus.^1^ Moreover, cholesterol content in the lens, one of the highest cholesterol containing tissues in the body, also increases with age.^26^ An analysis of lens phospholipids from different animal species found choline-containing phospholipids to be the most prevalent phospholipids, and in the bovine lens, phosphatidylcholine (PC) and sphingomyelin (SM) are the major classes.^27^ Consistent with these studies, we identified PCs and SMs as the most abundant classes of lipids noncovalently bound to bovine AQP0.

In human lenses, sphingolipid content increased with age whereas phosphatidylcholine content decreased.^27–29^ The ratio of sphingomyelin to other lens lipids is higher in the nucleus compared to the cortex and it has been proposed that the human lens contains high levels of sphingolipids to resist lipid oxidation, a detrimental consequence of aging.^29,30^ Given that the lipid composition of AQP containing membranes affects AQP water permeability,^15^ it is important to characterize the lipids interacting with AQPs in different lens regions.

Using native MS, we found that a population of tetrameric AQP0 isolated from both the cortex and nucleus regions of bovine lenses retained endogenous noncovalently bound lipids. The clusters of peaks at higher masses within the isolation window likely represent a mixture of modified forms of AQP0, as suggested by the number of lipids released from AQP0 complexes in MS/MS experiments. For example, in Figure 1A, three putative adduct peaks are labeled, +729, +810 and +893. It is possible that the +729 adduct peak represents AQP0 bound to PC 32:0 or PC 32:1 (predicted mass shifts of 733 or 731, respectively); lipids that dissociated upon MS/MS analysis. The +810 adduct peak could represent phosphorylated AQP0 with PC 32:0 or PC 32:1 adducted. Alternatively, the +810 peak could represent the *m*/*z* 813.8644 lipid found in inset Figure 1b and the +893 adduct peak could then represent phosphorylated AQP0 with this lipid. However, we suspect that these peaks represent a mixture of these proposed proteoforms.

We found that in the analysis of crude membranes (displayed in Figure 1 and Figure 2), AQP0 retained six of the same endogenous lipids, PC 16:0_16:0 (theoretical [M + H]^+^ 734.5694), PC 32:1 (theoretical [M + H]^+^ 732.5465), PC 34:1 (theoretical [M + H]^+^ 760.5894), PC 36:1 (theoretical [M + H]^+^ 788.6164), SM 40:2;2O (theoretical [M + H]^+^ 785.6531) and SM 42:2;2O (theoretical [M + H]^+^ 813.6844). However, AQP0 from the cortical region appeared to bind two different PCs (PC 30:0 (theoretical [M + H]^+^ 706.5381) and PC 36:4 (theoretical [M + H]^+^ 782.5694)) that were not bound to AQP0 from the nucleus. In contrast, three different SMs (SM 40:1;2O (theoretical [M + H]^+^ 787.6687), SM 42:3;2O (theoretical [M + H]^+^ 811.6687), and SM 44:2;2O (theoretical [M + H]^+^ 841.7157) were detected bound to AQP0 from the nucleus that were not bound to cortical AQP0. Supplementary Figure 33 contains a Venn diagram comparing the differences and similarities between the AQP0 bound lipids from the cortex and nucleus. In line with the literature, several of the lipids detected in this study were previously reported in the lens via MALDI-TOF-MS^31^ and ESI-MS/MS.^27^ Thus, there appears to be regional differences in the lipid environment in which AQP0 resides. Specifically, AQP0 in the nucleus is surrounded by a greater variety of SMs compared to the cortex. The functional differences in AQP0 water permeability in different lens regions have not been measured since early studies on mouse lenses only reported cortical lens water permeability.^32^

The sample preparation process in this study is delipidating by nature^33^ so, some noncovalently bound lipids to AQP0 may have been removed. Therefore, we incubated purified AQP0 first with a single lipid (DPPC) and subsequently with a native lens lipid extract from the cortex and nucleus to confirm DPPC binding and to uncover new AQP0-lipid interactions not detected in our crude samples.

As expected, purified AQP0 bound exogenous DPPC which allowed us to further confirm the identity of one of the most abundant peaks (*m*/*z* 734). Interestingly, purified AQP0 bound an assortment of lipids from both a cortical and nucleus lens lipid extract. AQP0 bound less of a variety of lipids from the cortical lens lipid extract which may be because the lens cortical tissue region is smaller compared to the nucleus and therefore there is likely a large difference in lipid abundances between the two regions. It is noteworthy that the lens lipid extracts contain a couple hundred lens lipids and thus the observation that AQP0 still binds many of the same lipids in both types of experiments (crude AQP0 vs purified AQP0 with incubated lens lipid extract) as seen in Supplementary Figure 33 suggests that AQP0 may preferentially bind certain lipids over others. Moreover, AQP0 bound five different ether-linked PCs from the nucleus extract. These ether-linked PCs were not detected in the crude AQP0 experiments and perhaps were not in direct contact with AQP0 and/or were weakly bound and were thus washed away during the sample preparation process. Deeley et al.,^34^ identified alkyl ether glycerophospholipids in the human lens specifically 1-*O*-alkyl glycerophosphoethanolamines and 1-*O*-alkyl glycerophosphoserines.

It is important to note that some weakly bound lipids may not have been detected due to dissociation during transmission through the mass spectrometer producing weak signal. Moreover, while all data were collected in positive-ion mode, it is known that certain lipid classes ionize better in negative-ion mode. Lastly, given the abundance and variety of lipids in the crude samples, background lipids could be isolated along with AQP0-lipid complexes. However, when extracted lens lipids were analyzed in the absence of AQP0, tandem mass spectra from the same isolated region of the mass spectrum showed no lipid signals (data not shown).

## Conclusion

Sanders and Mittendorf^35^ reported that many membrane proteins have evolved to withstand changes in membrane lipid composition, and cited AQP0 as an exemplary protein that does not display lipid specificity. Based on high-resolution structures, AQP0, in different lipid bilayers, adopts quite similar conformations suggesting AQP0 structure, and therefore function, is not dictated by specific lipid binding.^35^ Conversely, functional studies established that AQP0 and AQP4 water permeability is dependent on membrane lipid composition.^15,36^ Specifically, Tong et al.,^15^ reported that AQP0 unit channel permeability was lower in bilayers containing higher levels of cholesterol and sphingomyelin. So, while AQP0 remains functional as a water channel in diverse lipid environments, it is activity, or water permeability, is dependent on specific membrane lipid compositions. Our study reveals that, physiologically, AQP0 is surrounded by a complex lipid matrix that may, dynamically, regulate AQP0 function in a differential manner across the lens. The results from this study can be used to inform functional and structural experiments that seek to gain a better understanding of AQP0 in the context of its native environment and, further, its role in the lens microcirculation system.

## Supplementary Material



## Notes

Tandem mass spectra for all identified lipids, extended mass spectra of tetrameric AQP0, extended tandem mass spectra of tetrameric AQP0, Venn diagram comparing AQP0-bound lipids, and calculated mass error of LC-MS/MS identified lipids (PDF)
