# Supplementary material for: Endogenous Aquaporin‑0 Lipid Binding in Ocular Lens Tissue via Native Mass Spectrometry
Source: J Am Soc Mass Spectrom. 2025 Jun 24;36(8):1588–97. doi: 10.1021/jasms.4c00500 (PMC12333349; doi:10.1021/jasms.4c00500)
Supplement: Supplementary file 1 [file js4c00500_si_001.pdf]

Supporting Information

Endogenous Aquaporin-0 Lipid Binding in Ocular Lens Tissue via Native Mass Spectrometry

Carla V.T. O'Neale[1], Sophie R. Harvey[2], Sergei Chetyrkin[3], Vicki H. Wysocki[4], Kevin L. Schey[1]*

1. Department of Biochemistry, Vanderbilt University, Nashville, TN, 37205
2. Department of Chemistry and Biochemistry and Native Mass Spectrometry Guided Structural Biology Center, The Ohio State University, Columbus, OH, 43210
3. Mass Spectrometry Research Center, Vanderbilt University, Nashville, TN, 37240
4. School of Chemistry and Biochemistry, Georgia Tech, Atlanta, GA, 30332

Corresponding Author: *Kevin L. Schey, PhD
Email: k.schey@vanderbilt.edu

Table of Contents

Supplementary Figures 1-21…………Tandem mass spectra for all identified lipids

Supplementary Figures 22-32………..Extended mass spectra and tandem mass spectra of tetrameric AQP0

Supplementary Figure 33……………..Venn-diagram comparing AQP0-bound lipids

Supplementary Tables 1-4……………..Calculated mass error of LC-MS/MS identified lipids.

PC 30:0

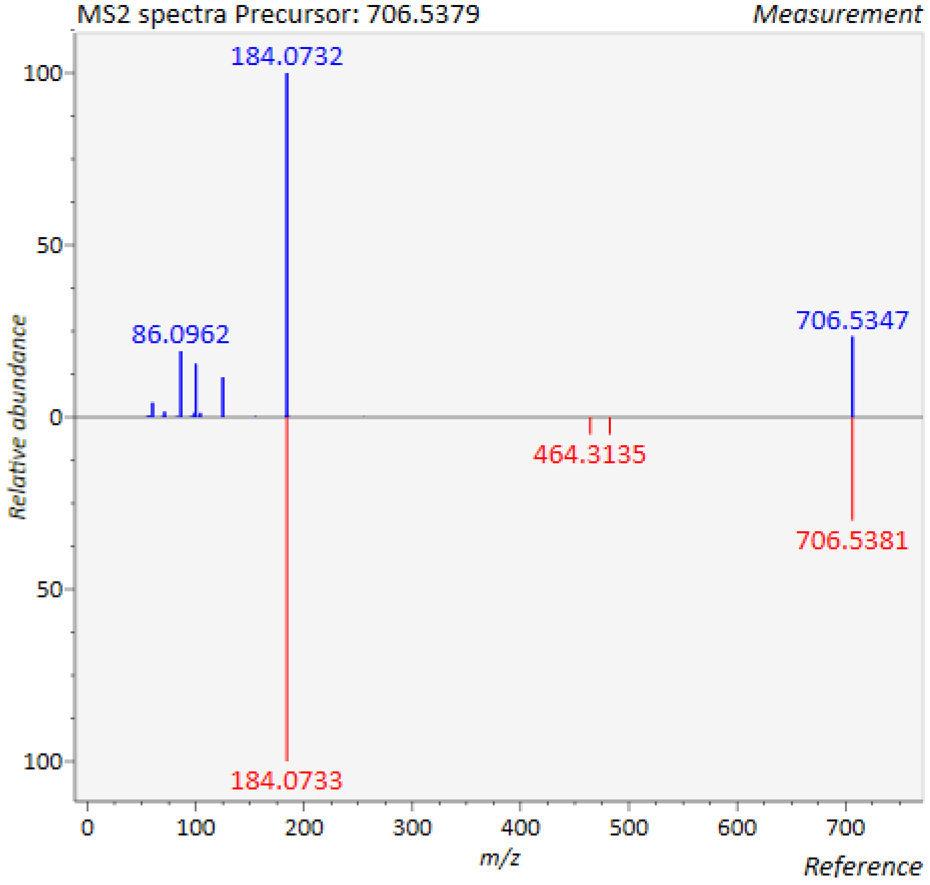

Supplementary Fig 1: PC 30:0 experimental (blue) and spectral library matching fragmentation spectra (red).

PC 32:1

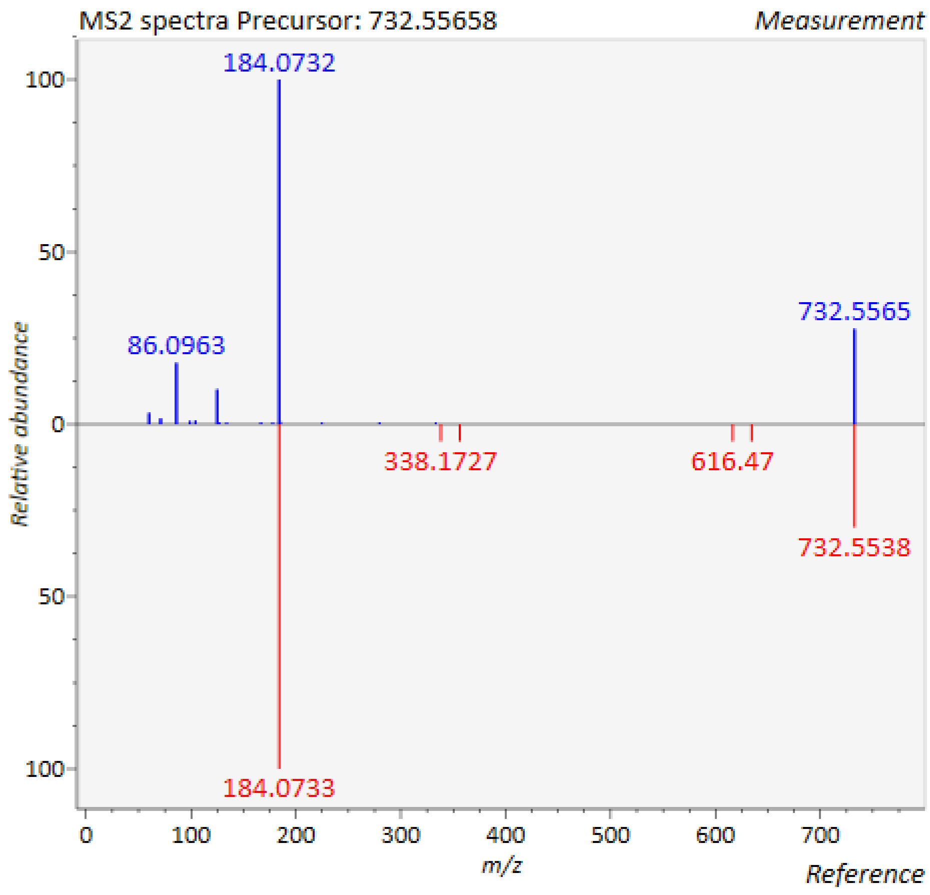

Supplementary Fig 2: PC 32:1 experimental (blue) and spectral library matching fragmentation spectra (red).

PC 16:0_16:0

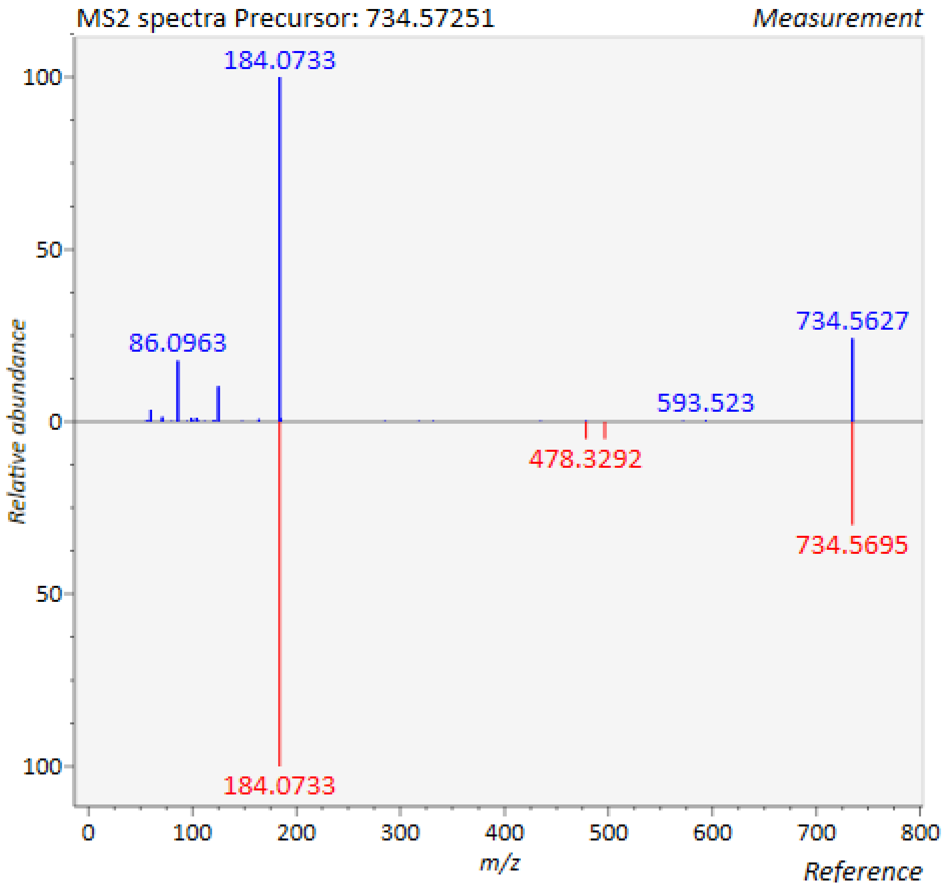

Supplementary Fig 3: PC 16:0_16:0 experimental (blue) and spectral library matching fragmentation spectra (red).

PC 34:1

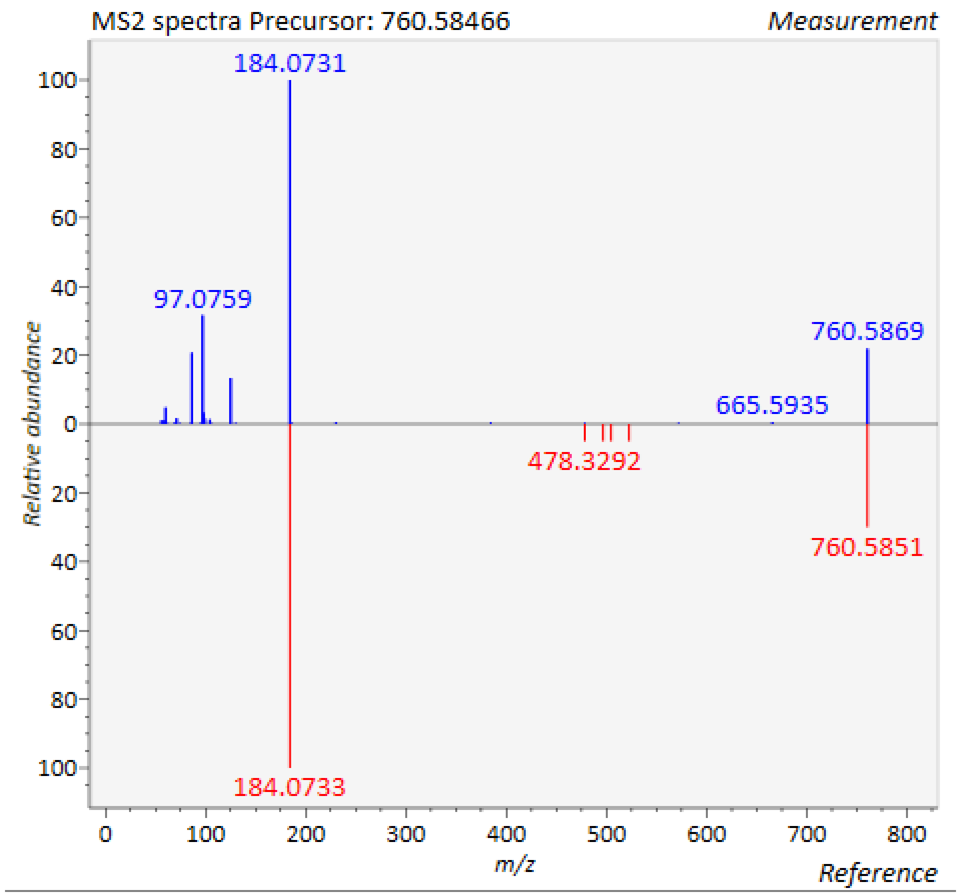

Supplementary Fig 4: PC 34:1 experimental (blue) and spectral library matching fragmentation spectra (red).

PC 36:4

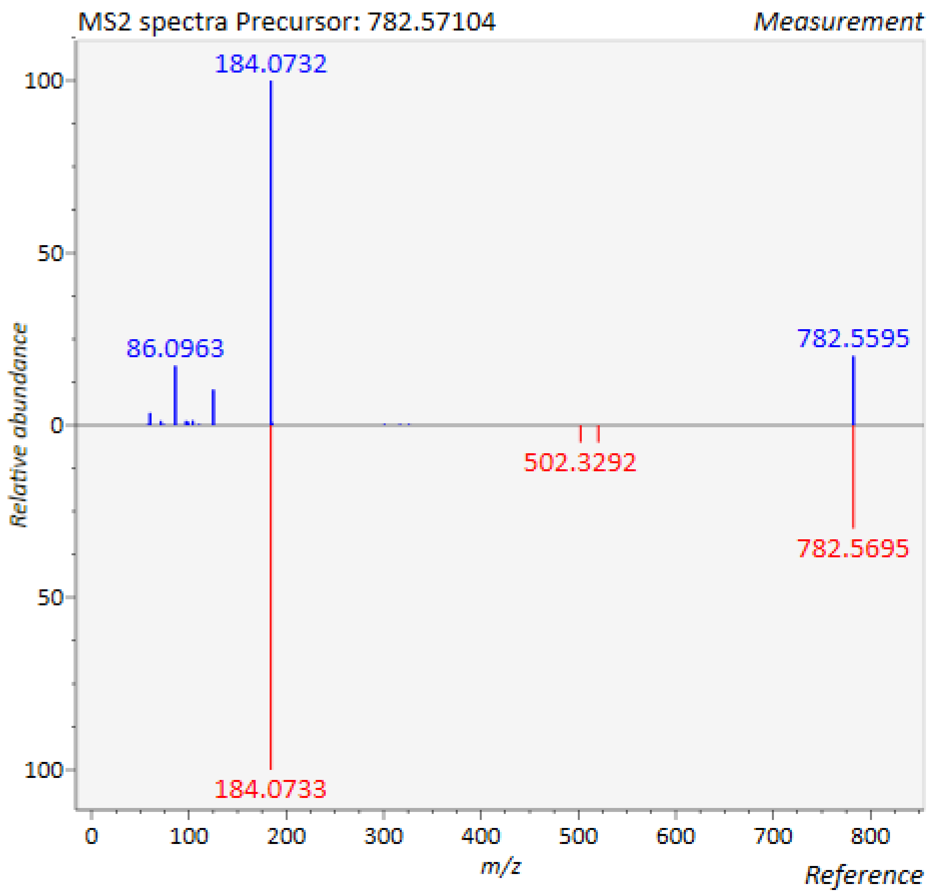

Supplementary Fig 5: PC 36:4 experimental (blue) and spectral library matching fragmentation spectra (red).

SM 40:1;2O

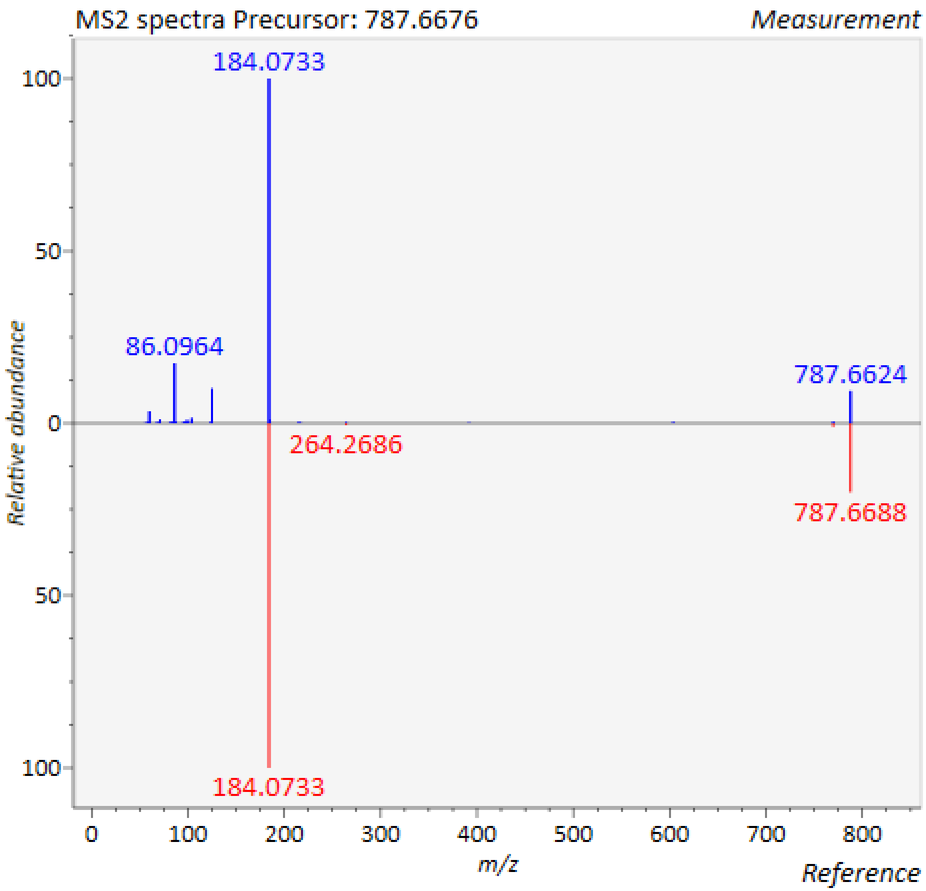

Supplementary Fig 6: SM 40:1;2O experimental (blue) and spectral library matching fragmentation spectra (red).

PC 36:2

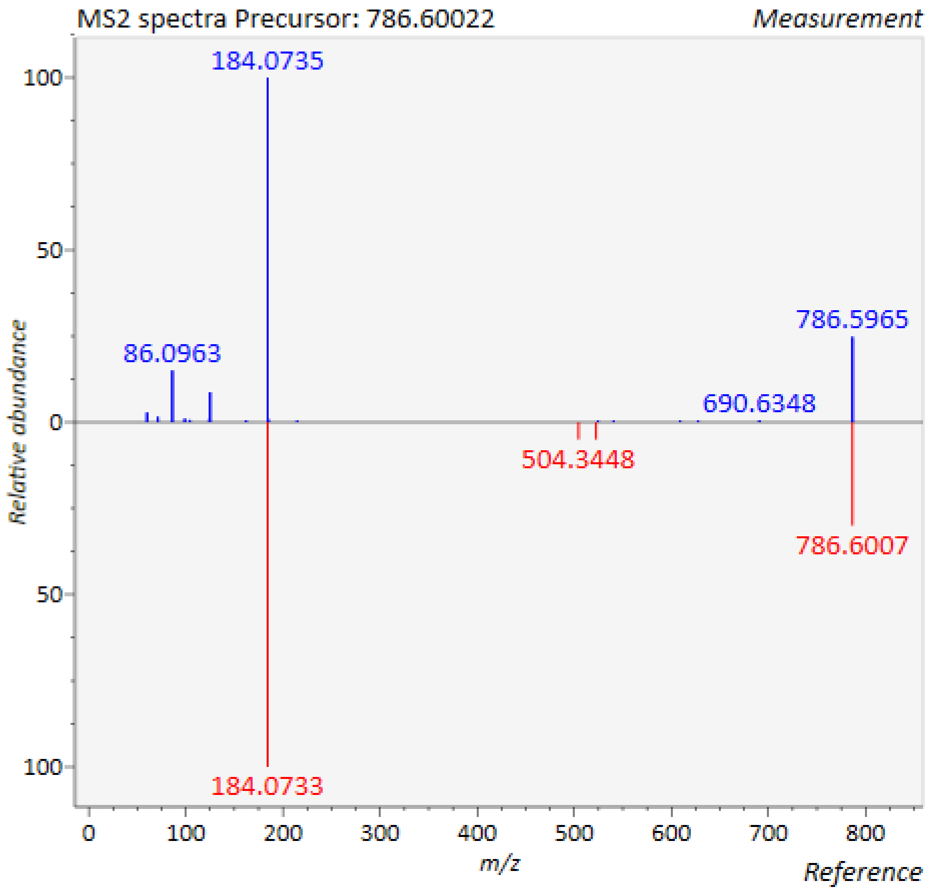

Supplementary Figure 7: PC 36:2 experimental (blue) and spectral library matching fragmentation spectra (red).

PC 33:0

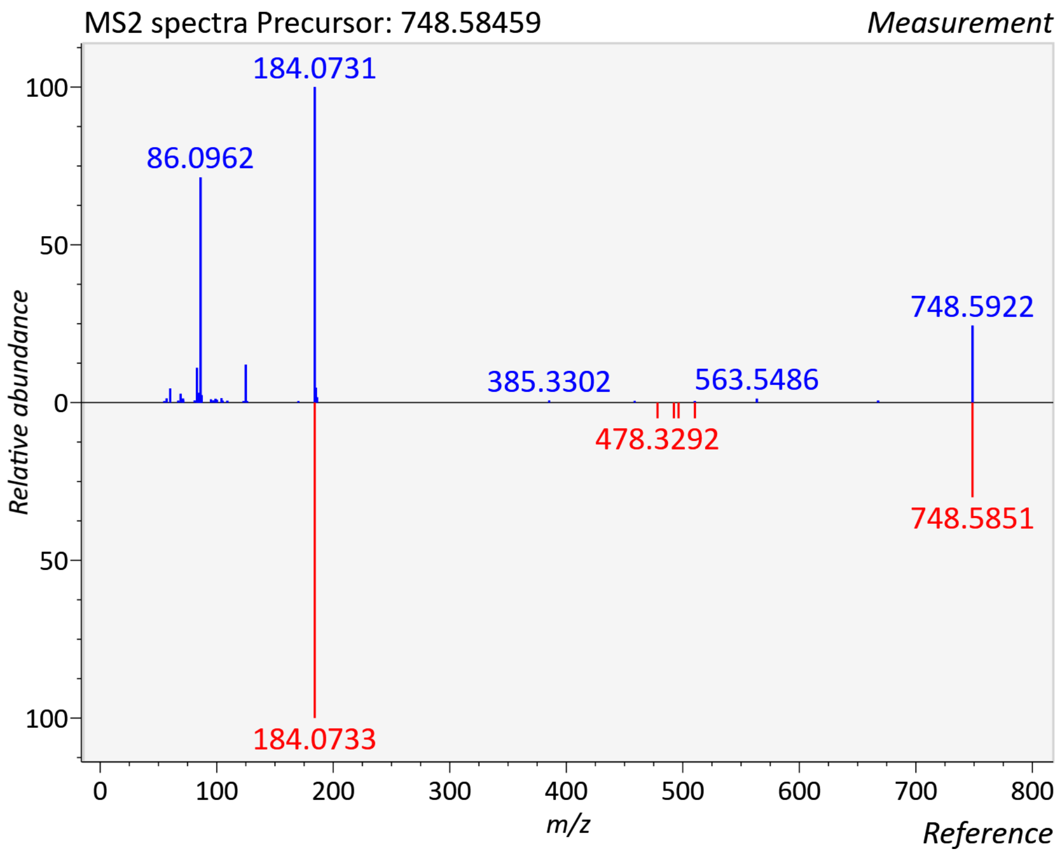

Supplementary Figure 8: PC 33:0 experimental (blue) and spectral library matching fragmentation spectra (red).

PC O-32:1

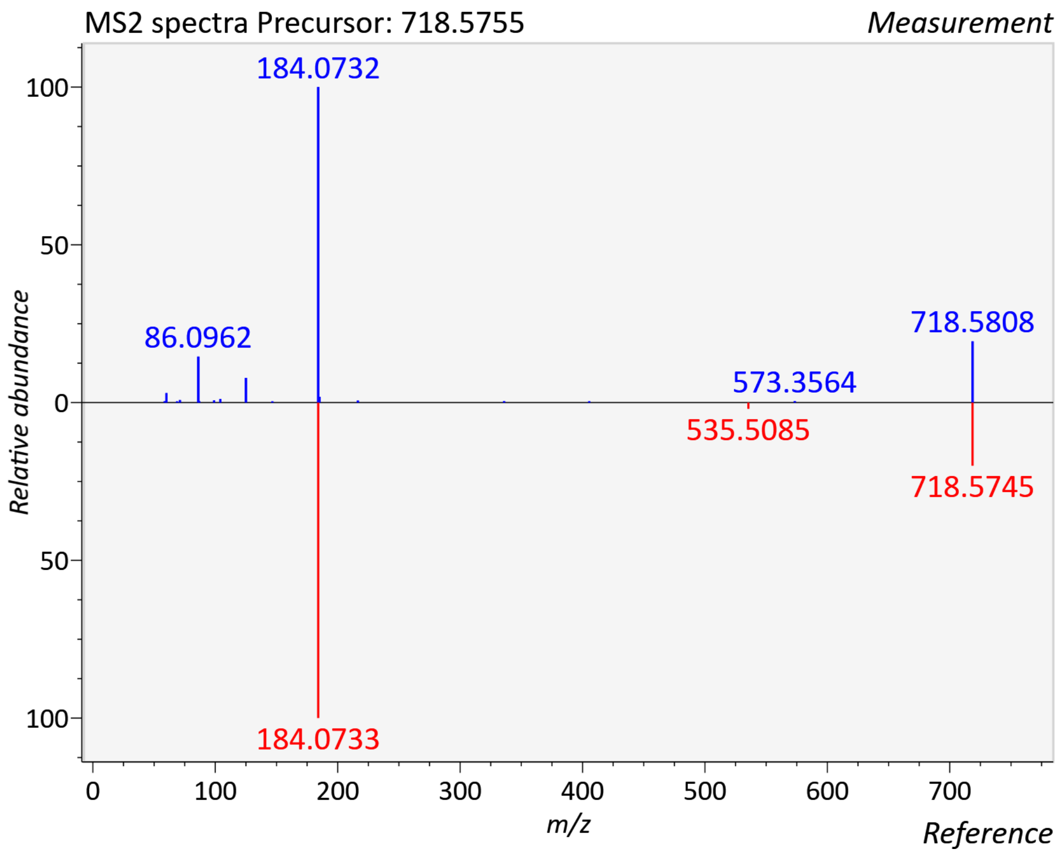

Supplementary Figure 9: PC O-32:1 experimental (blue) and spectral library matching fragmentation spectra (red).

PC O-30:0

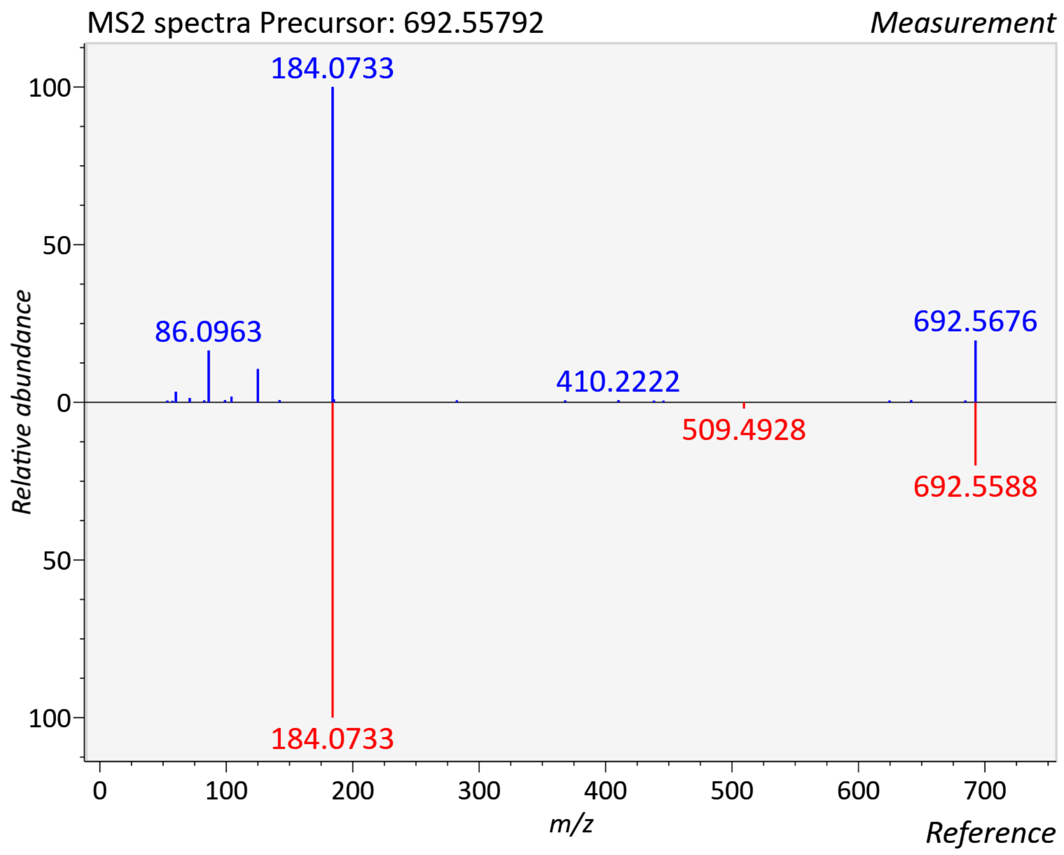

Supplementary Figure 10: PC O-30:0 experimental (blue) and spectral library matching fragmentation spectra (red).

PC O-32:0

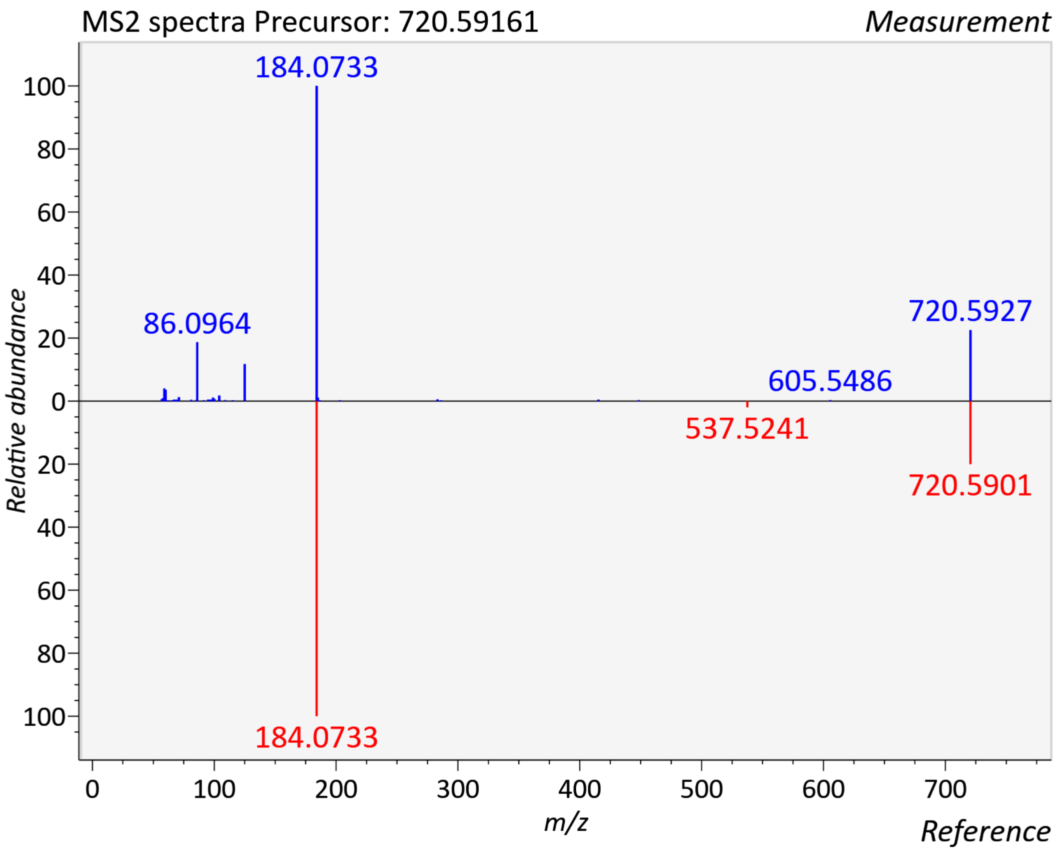

Supplementary Figure 11: PC O-32:0 experimental (blue) and spectral library matching fragmentation spectra (red).

PC O-34:2

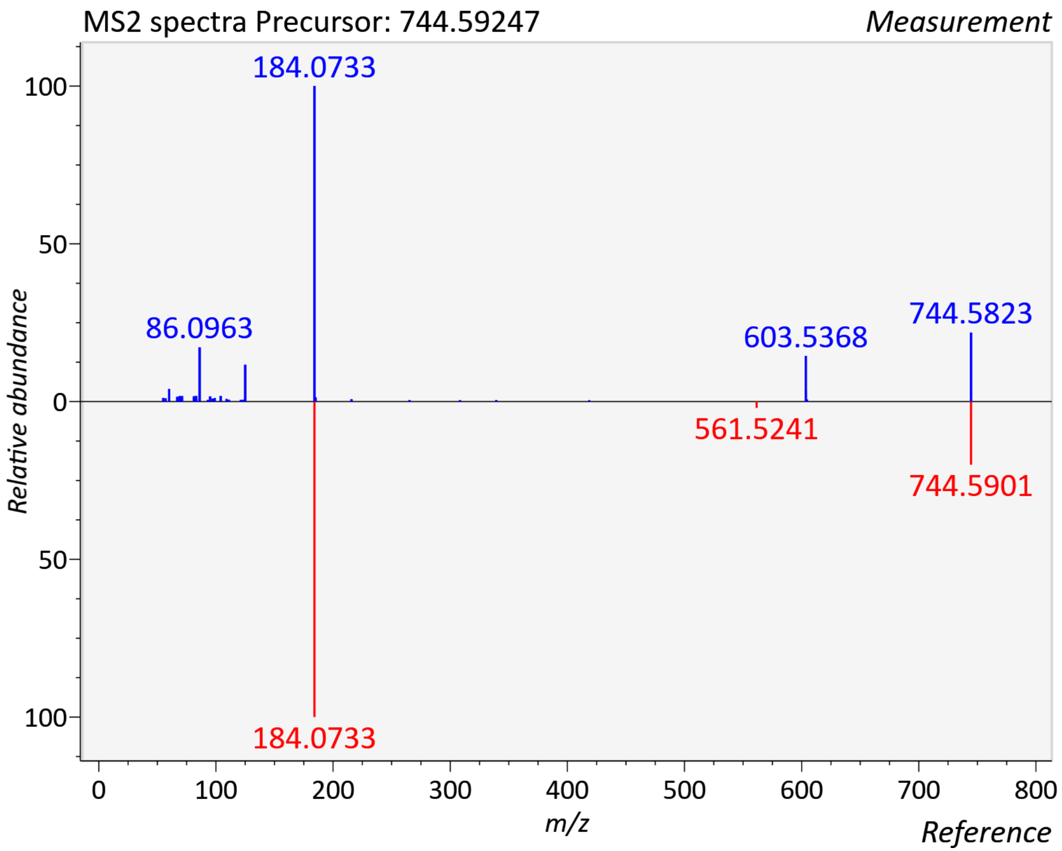

Supplementary Figure 12: PC O-34:2 experimental (blue) and spectral library matching fragmentation spectra (red).

PC O-34:1

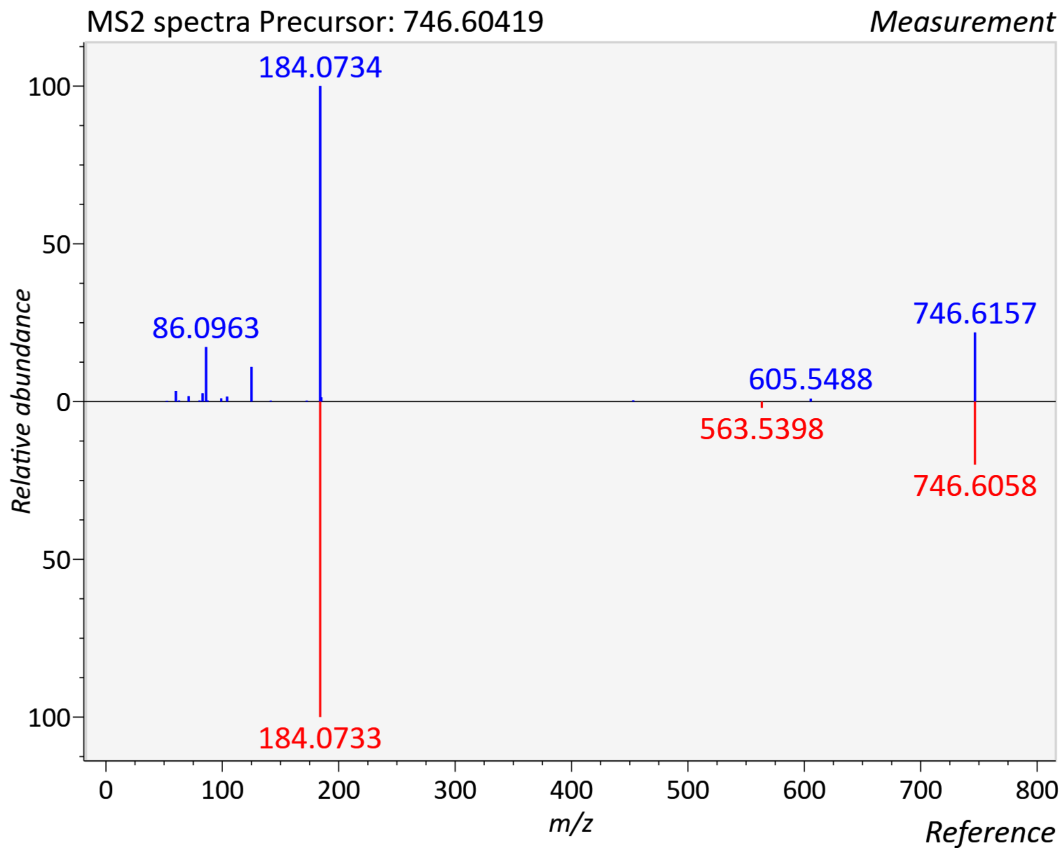

Supplementary Figure 13: PC O-34:1 experimental (blue) and spectral library matching fragmentation spectra (red).

SM 44:3;2O

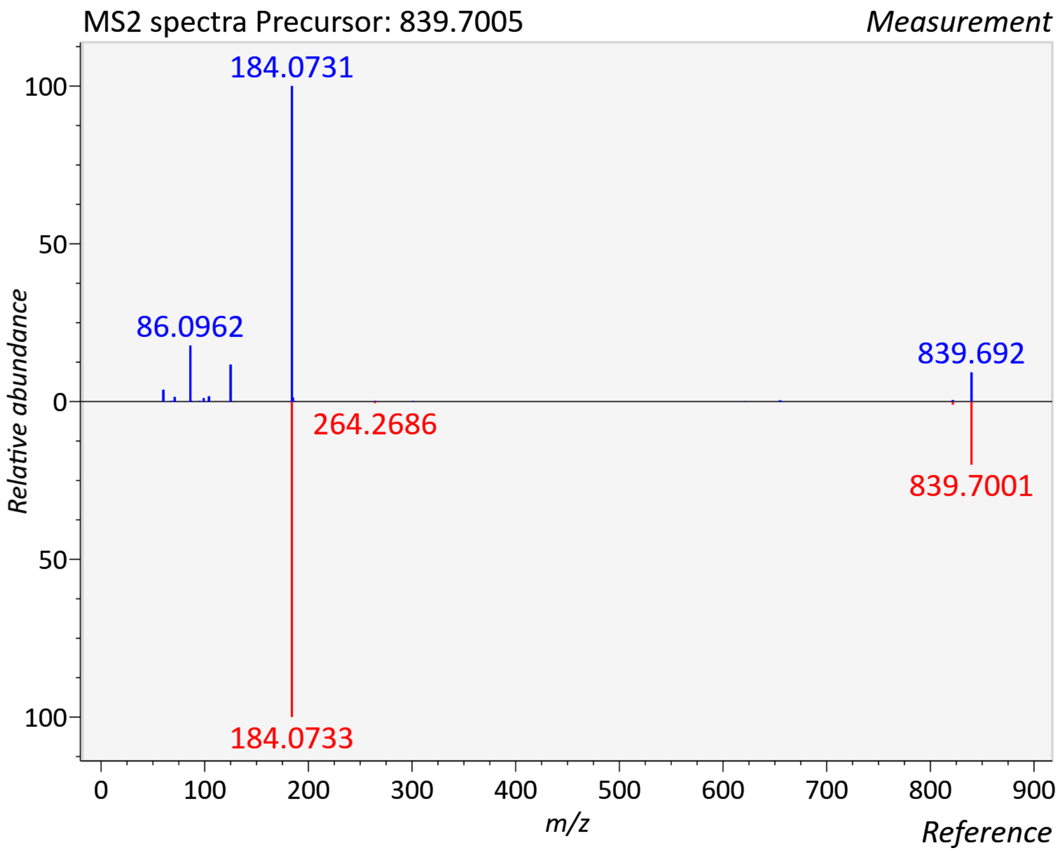

Supplementary Figure 14: SM 44:3;2O experimental (blue) and spectral library matching fragmentation spectra (red).

SM 41:2;2O

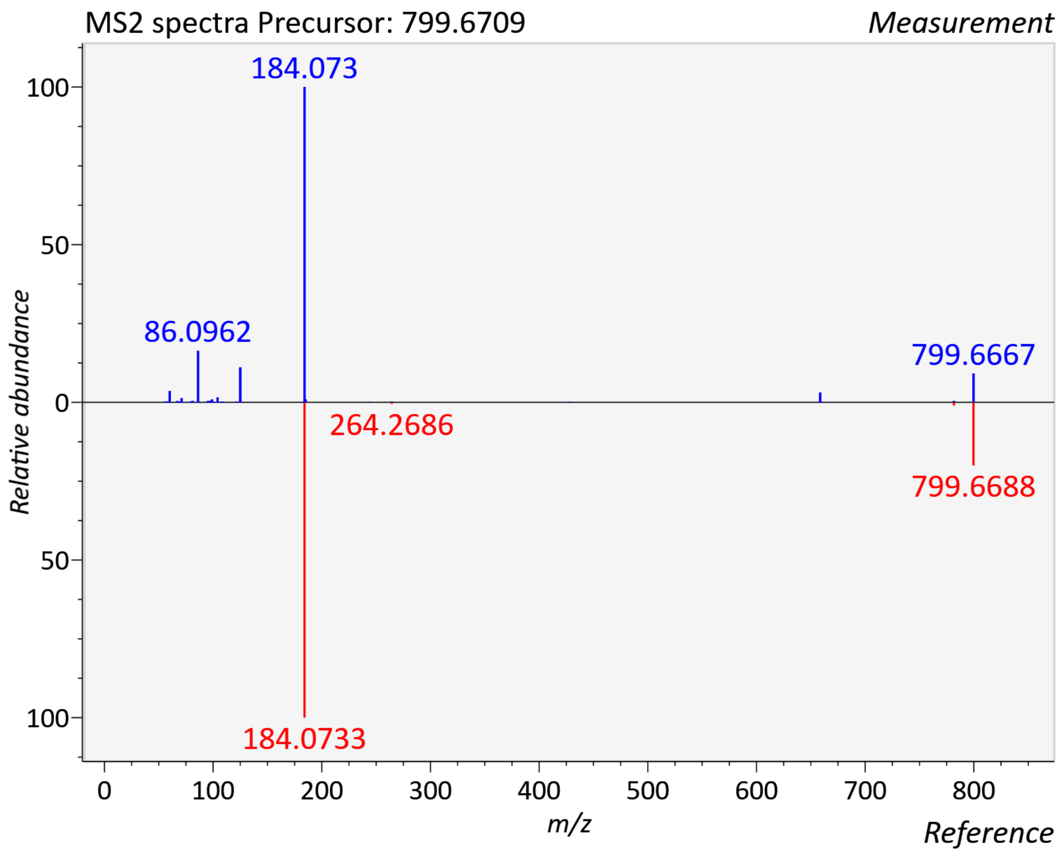

Supplementary Figure 15: SM 41:2;2O experimental (blue) and spectral library matching fragmentation spectra (red).

PC 36:1

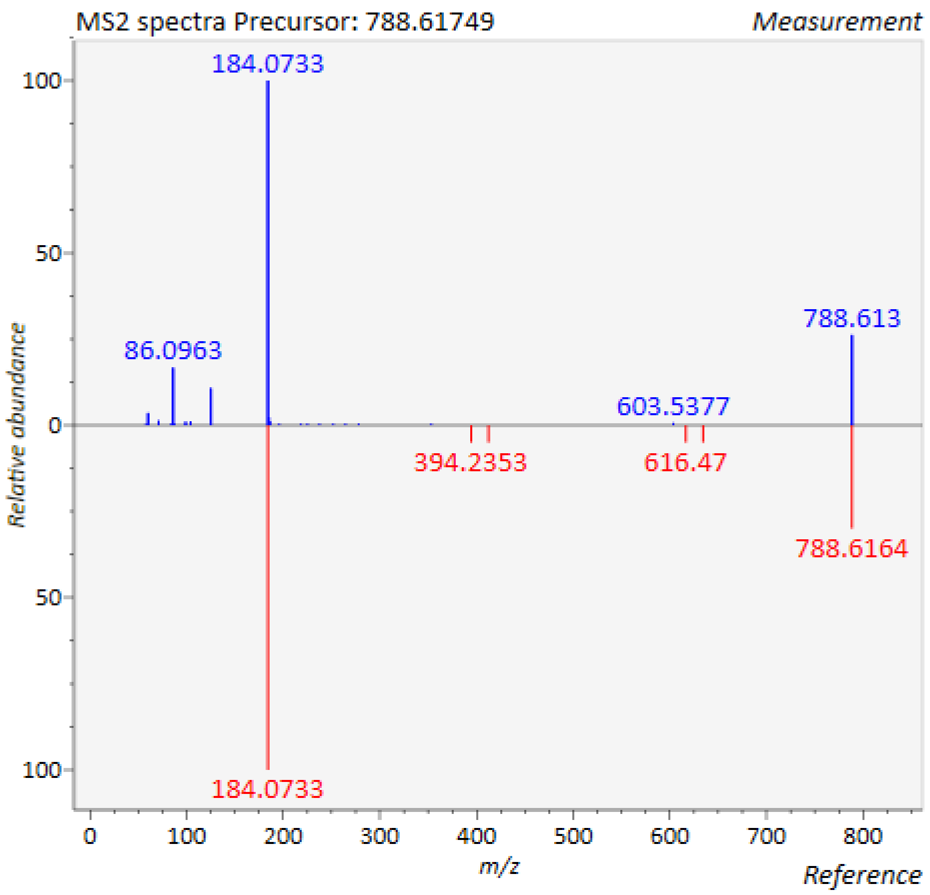

Supplementary Fig 16: PC 36:1 experimental (blue) and spectral library matching fragmentation spectra (red).

SM 42:3;2O

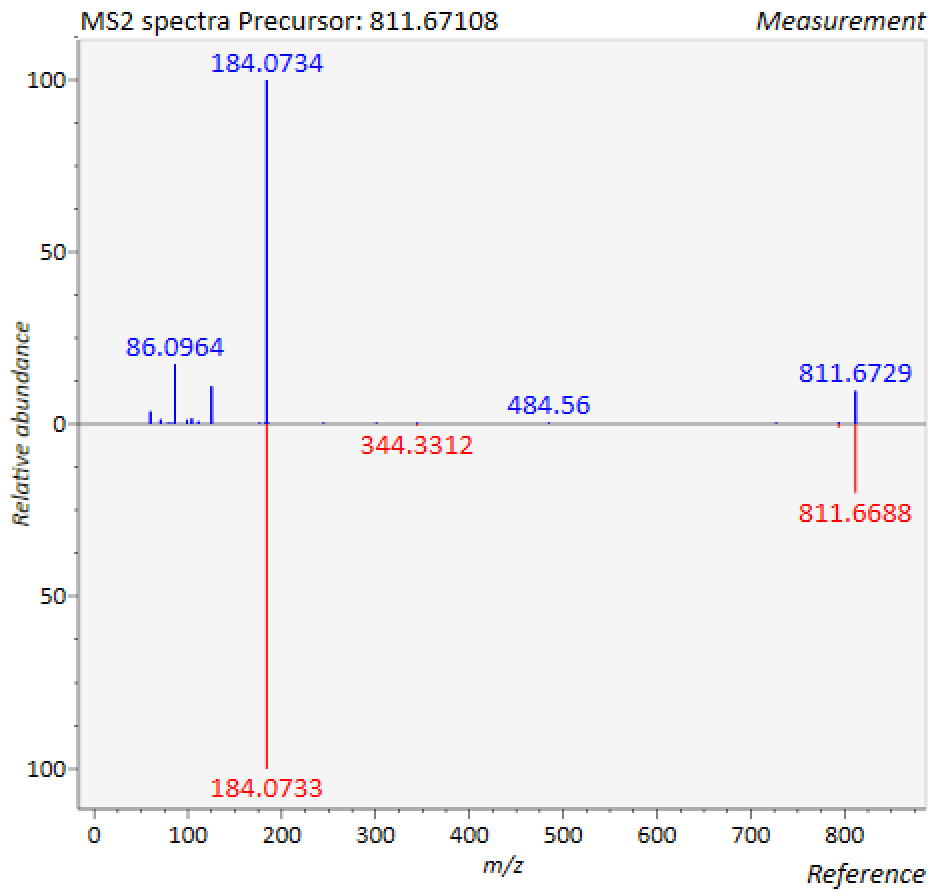

Supplementary Fig 17: SM 42:3;2O experimental (blue) and spectral library matching fragmentation spectra (red).

SM 42:2;2O

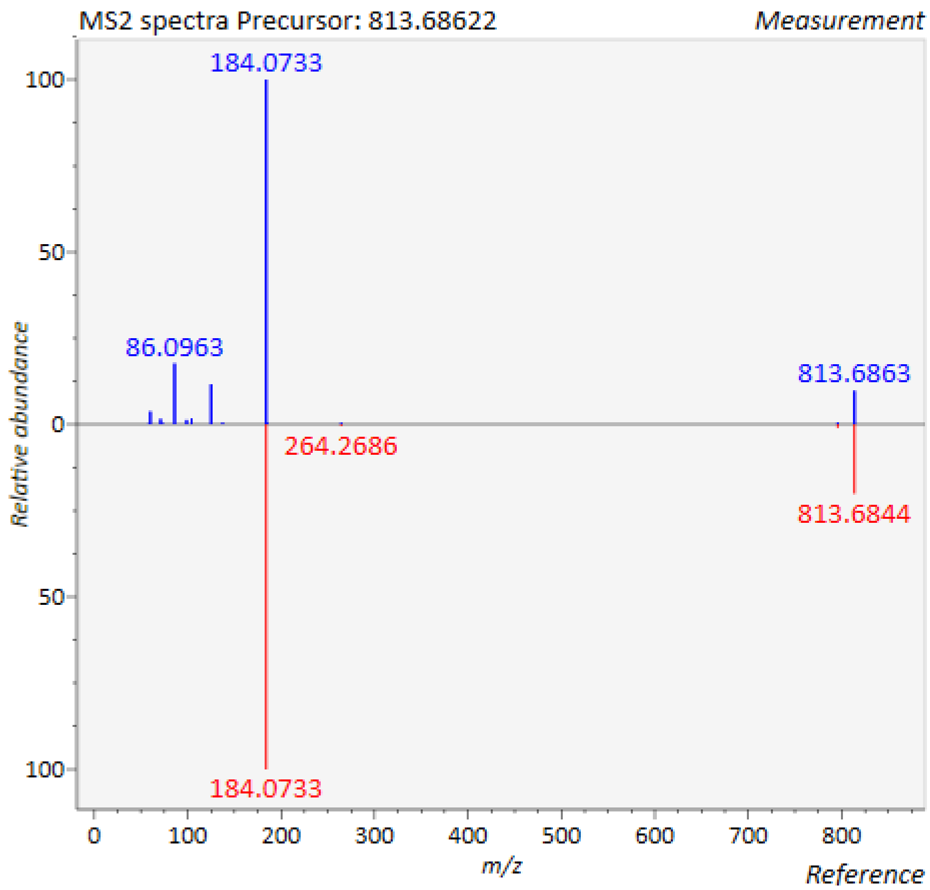

Supplementary Fig 18: SM 42:2;2O experimental (blue) and spectral library matching fragmentation spectra (red).

SM 44:2;2O

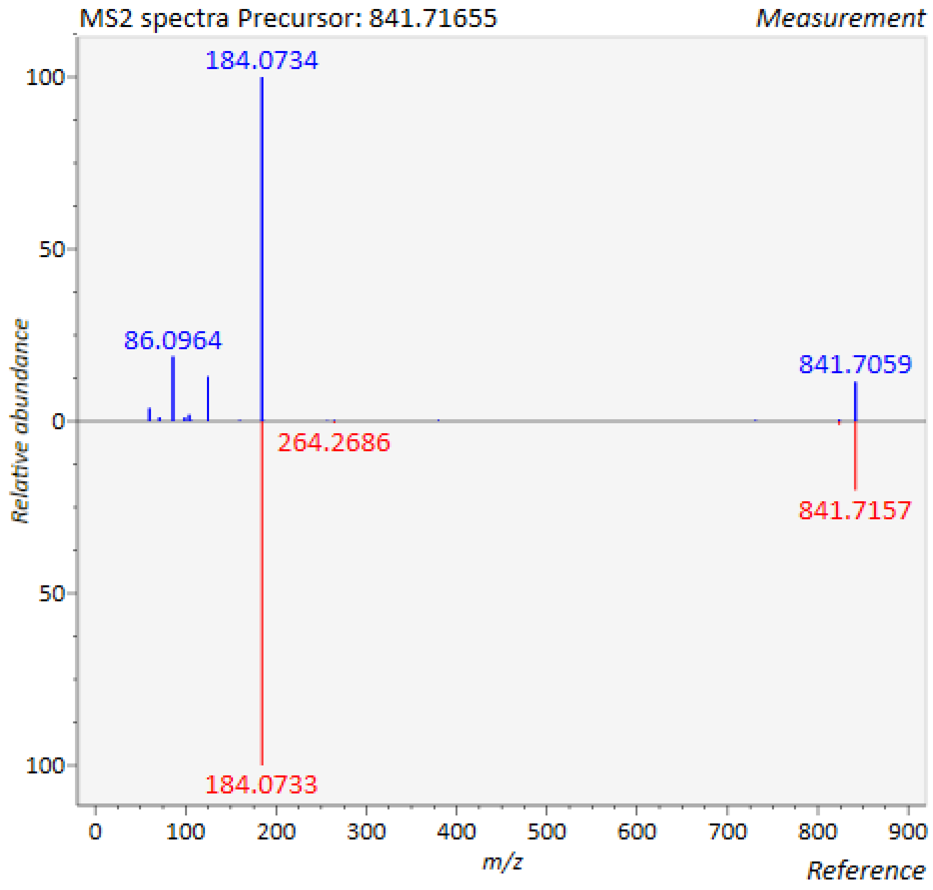

Supplementary Fig 19: SM 44:2;2O experimental (blue) and spectral library matching fragmentation spectra (red).

SM 40:2;2O

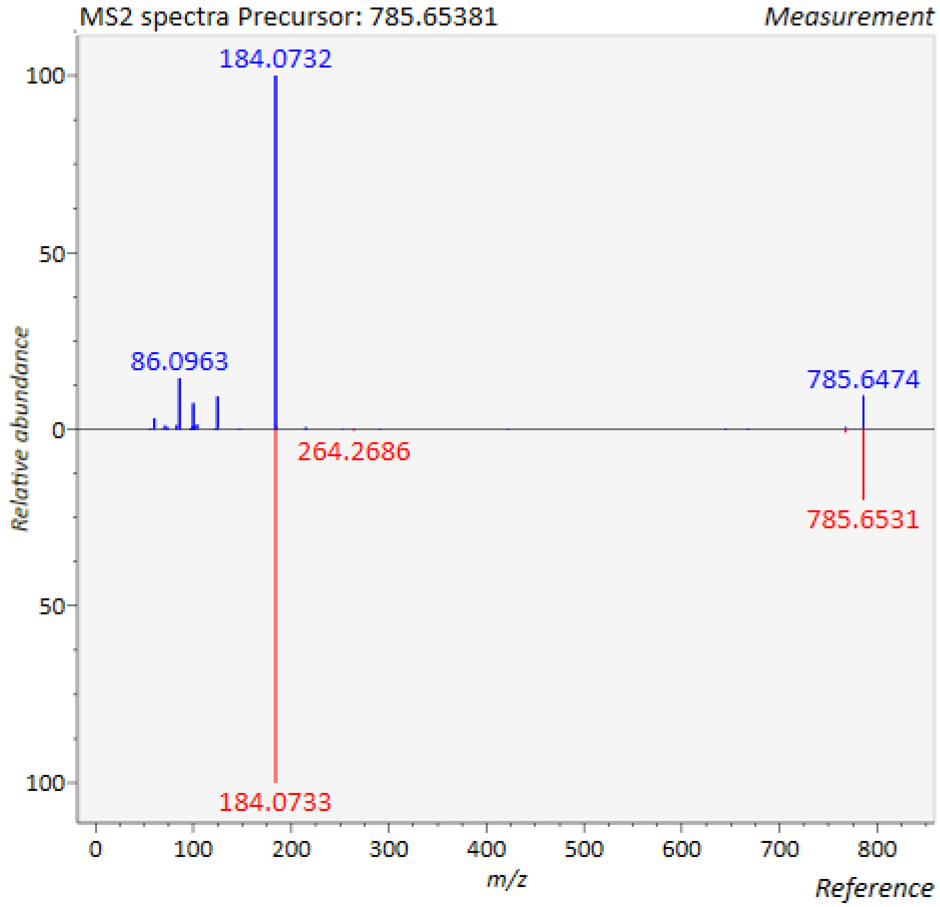

Supplementary Fig 20: SM 40:2;2O experimental (blue) and spectral library matching fragmentation spectra (red).

SM 34:1;2O

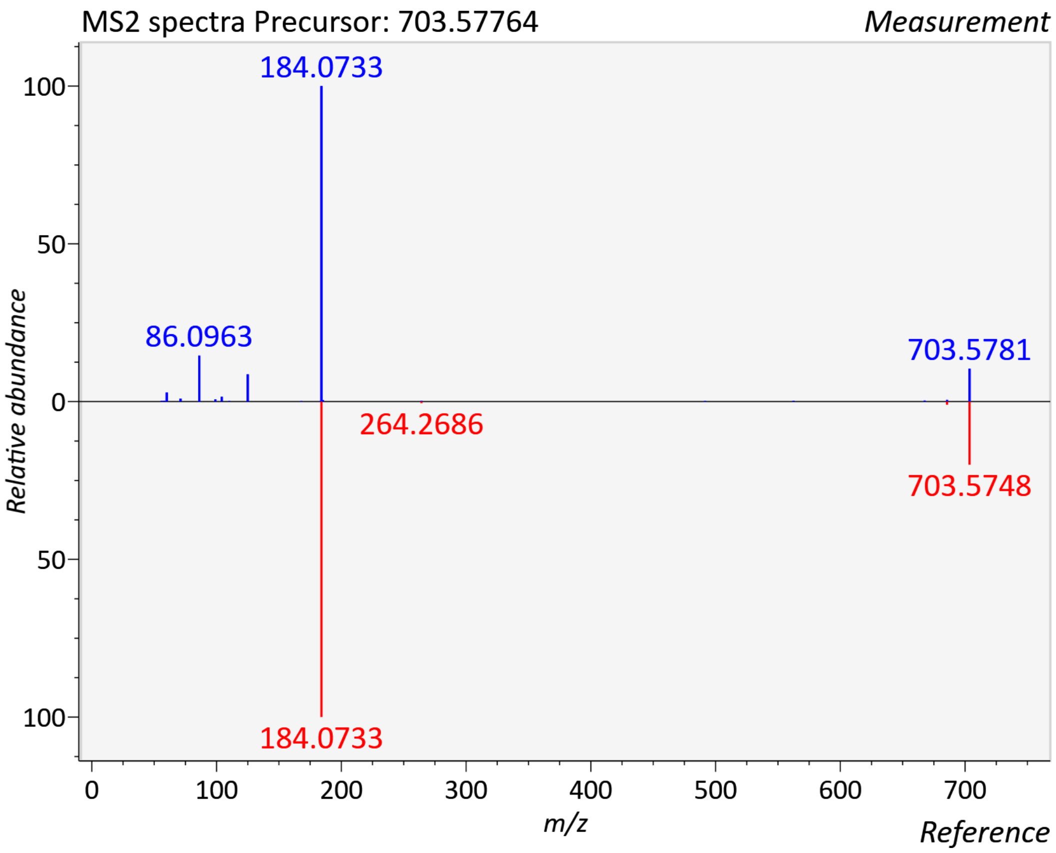

Supplementary Figure 21: SM 34:1;2O experimental (blue) and spectral library matching fragmentation spectra (red).

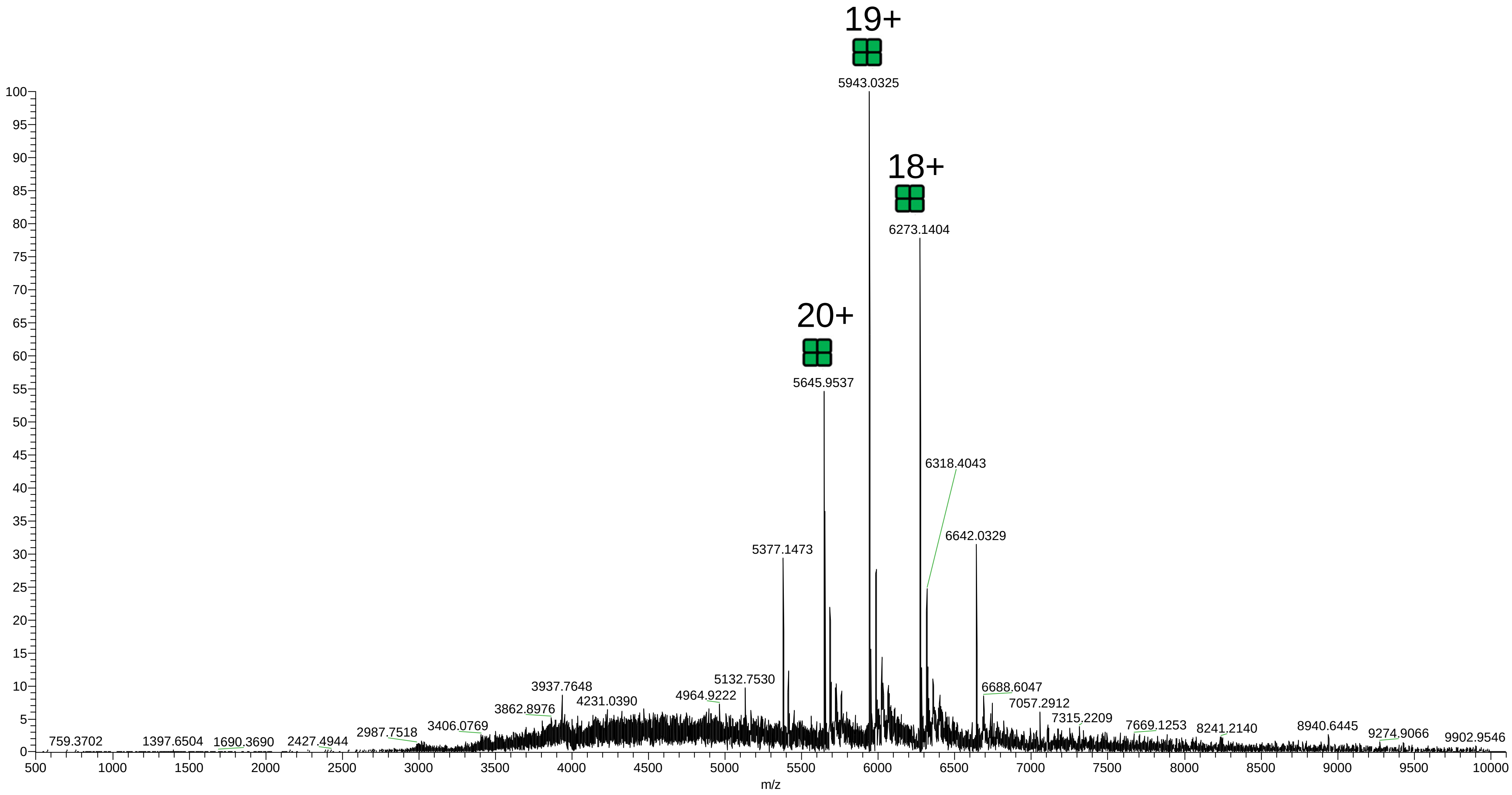

Supplementary Figure 22: Extended mass spectrum of tetrameric crude AQP0 from the bovine lens cortex.

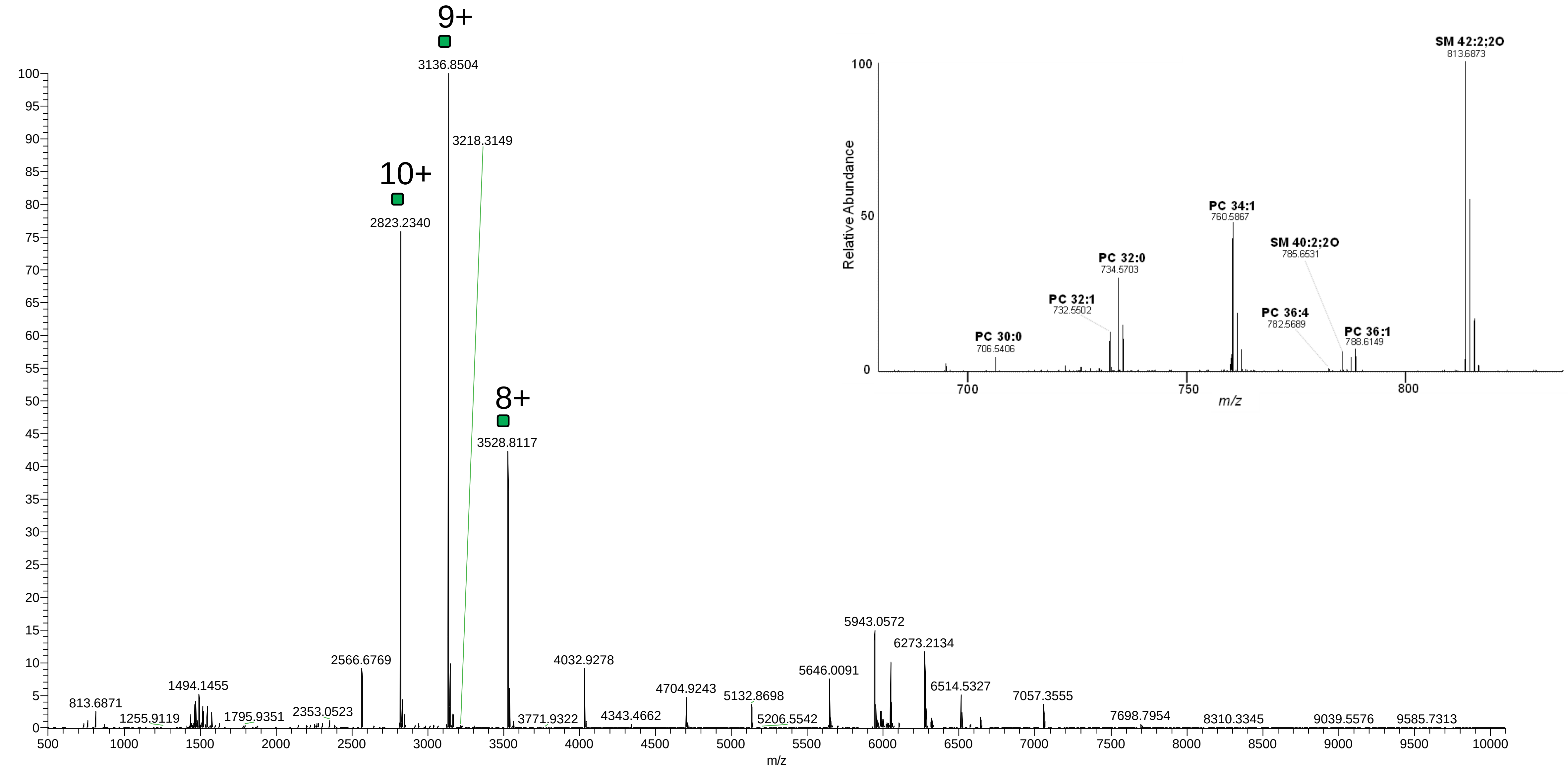

Supplementary Figure 23: Tandem mass spectrum of crude AQP0 from the bovine lens cortex after fragmentation at 200V of the isolation window highlighted in Figure 1A. Inset is a zoom-in of the low *m/z* range.

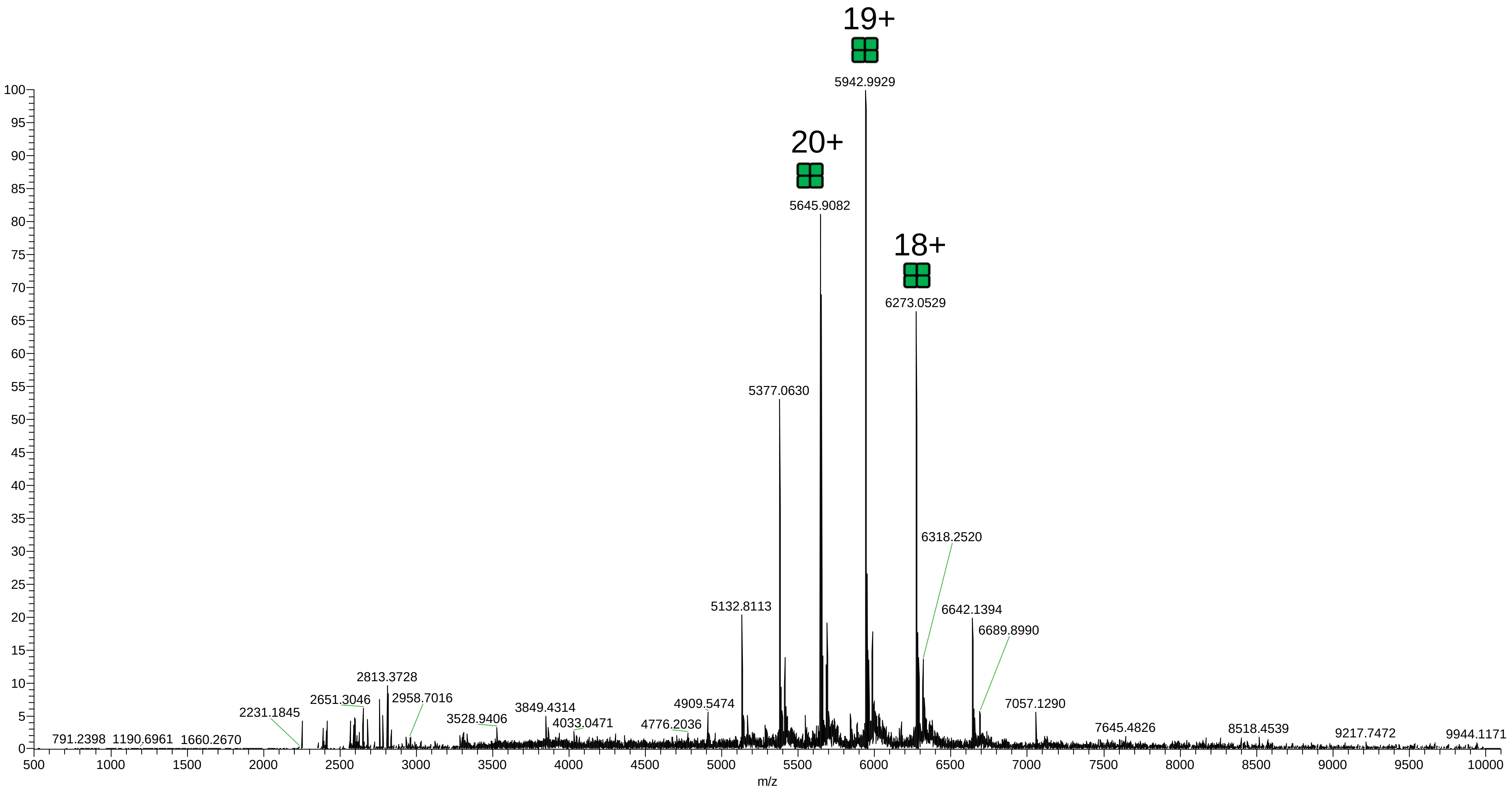

Supplementary Figure 24: Extended mass spectrum of tetrameric crude AQP0 from the bovine lens nucleus.

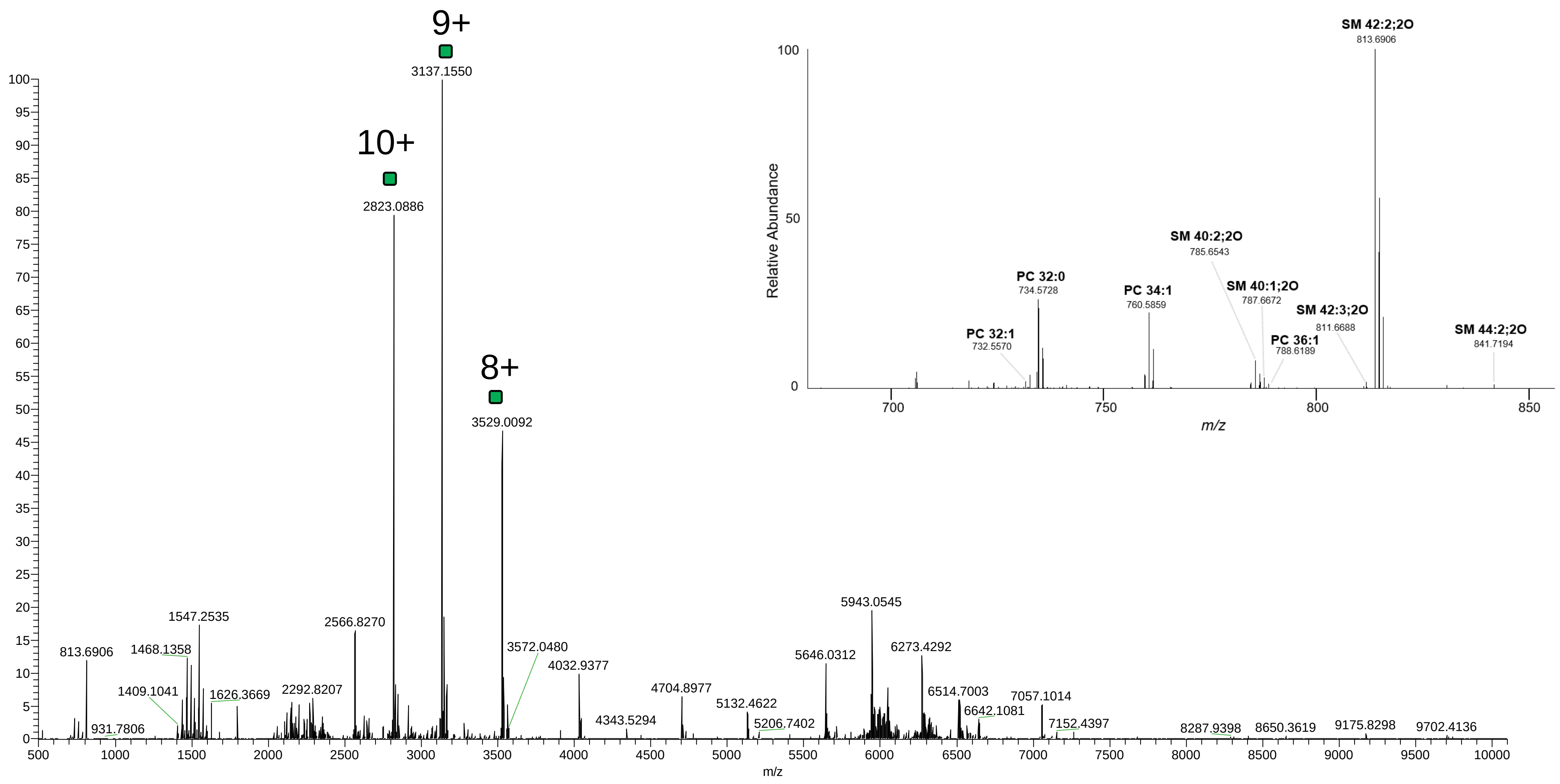

Supplementary Figure 25: Tandem mass spectrum of crude AQP0 from the bovine lens nucleus after fragmentation at 200V of the isolation window highlighted in Figure 2A. Inset is a zoom-in of the low *m/z* range.

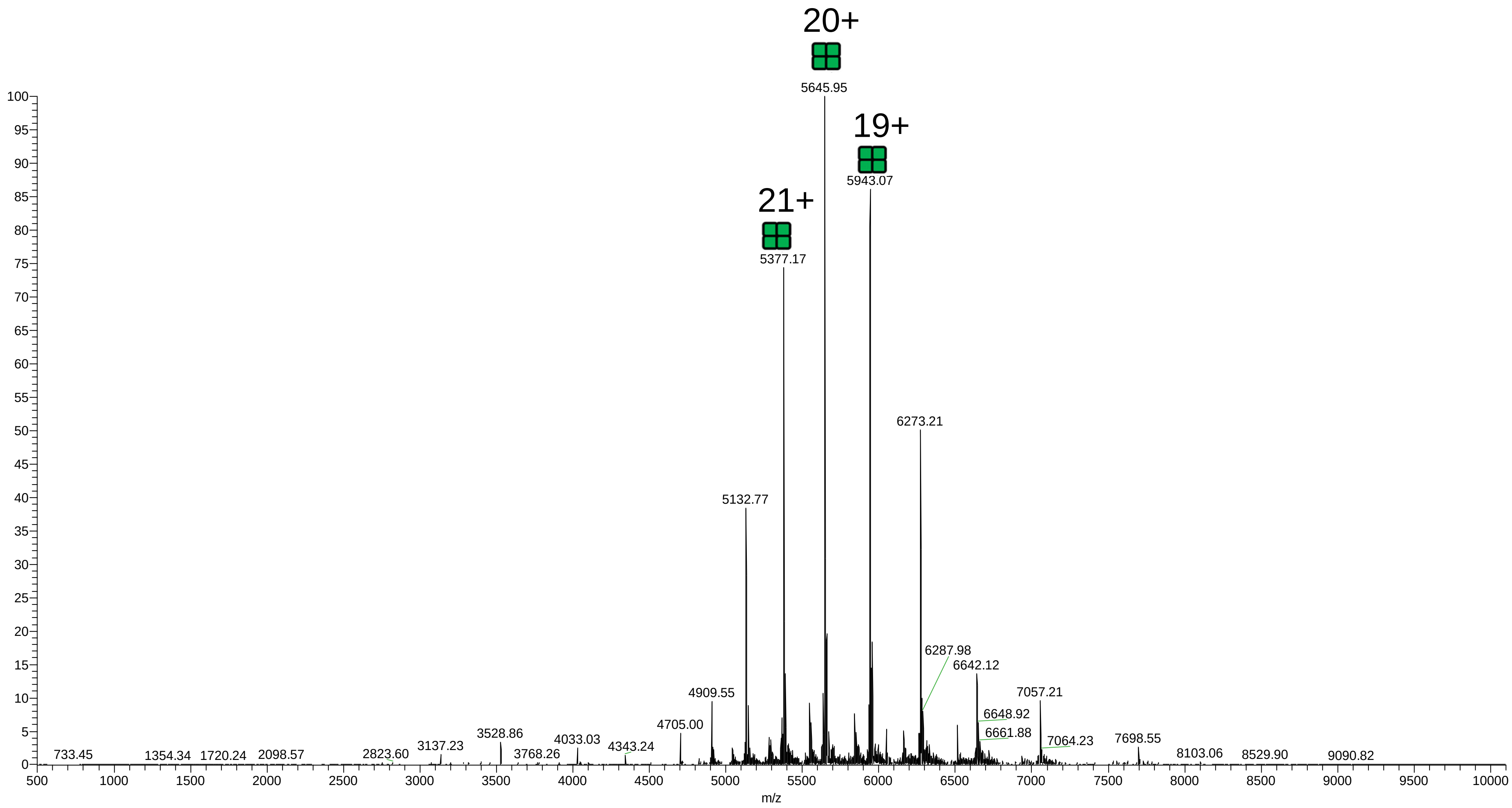

Supplementary Figure 26: Extended mass spectrum of purified tetrameric AQP0.

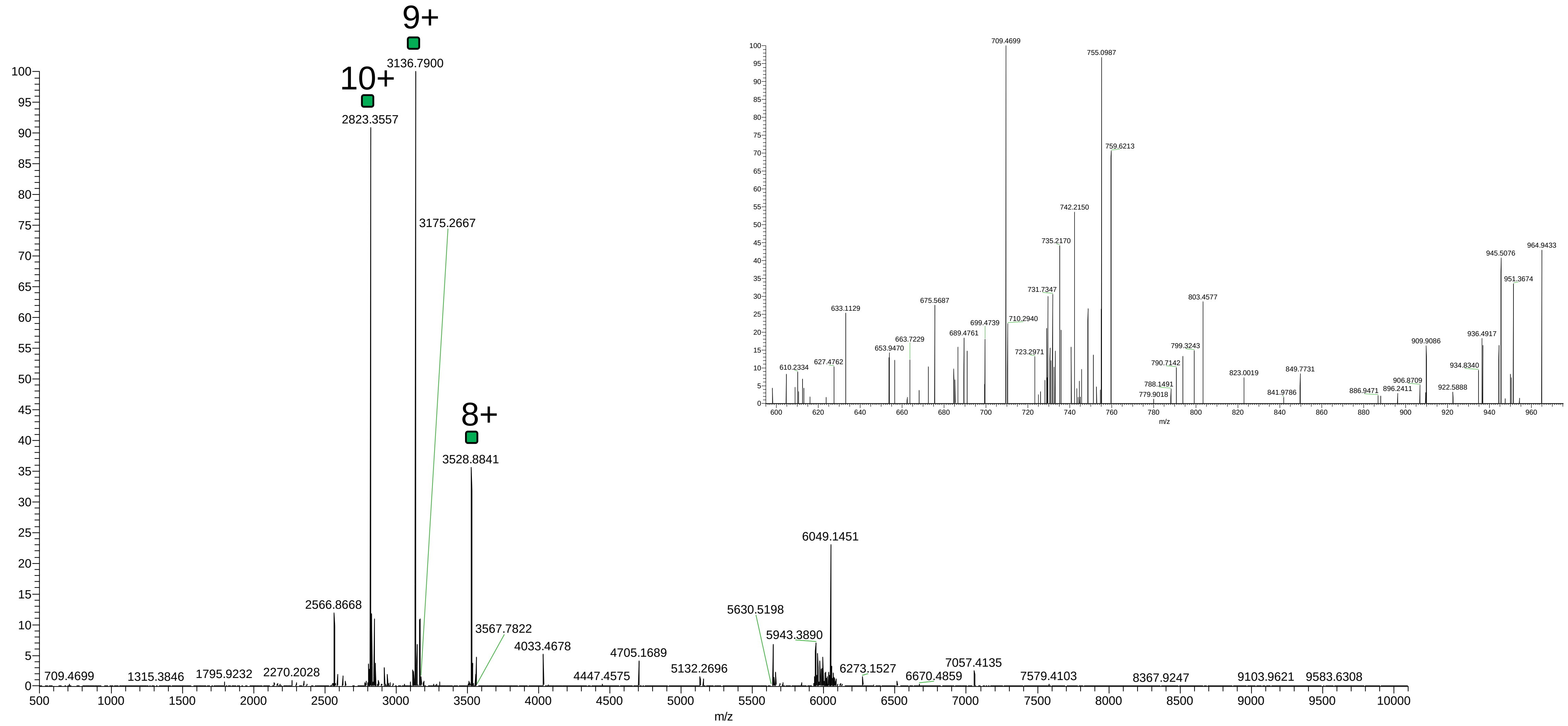

Supplementary Figure 27: Tandem mass spectrum of purified AQP0 after fragmentation at 200V of the 19+ charge state. Inset is a zoom-in of the low *m/z* range.

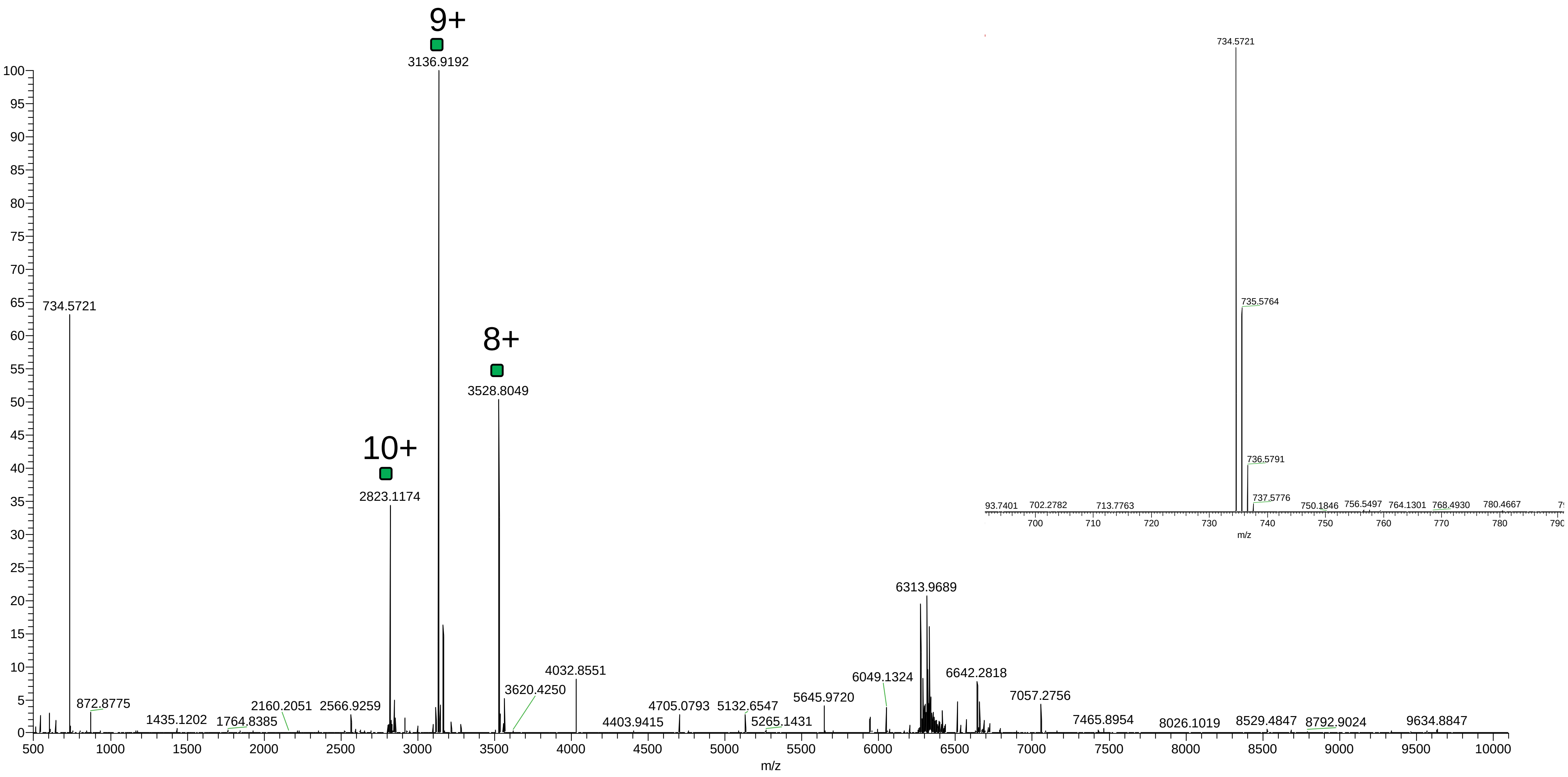

Supplementary Figure 28: Tandem mass spectrum of purified AQP0 with exogenous DPPC spiked-in. Inset shows zoom-in of DPPC lipid (*m/z* 734.5721) released from AQP0 after fragmentation at 200V of the 19+ charge state.

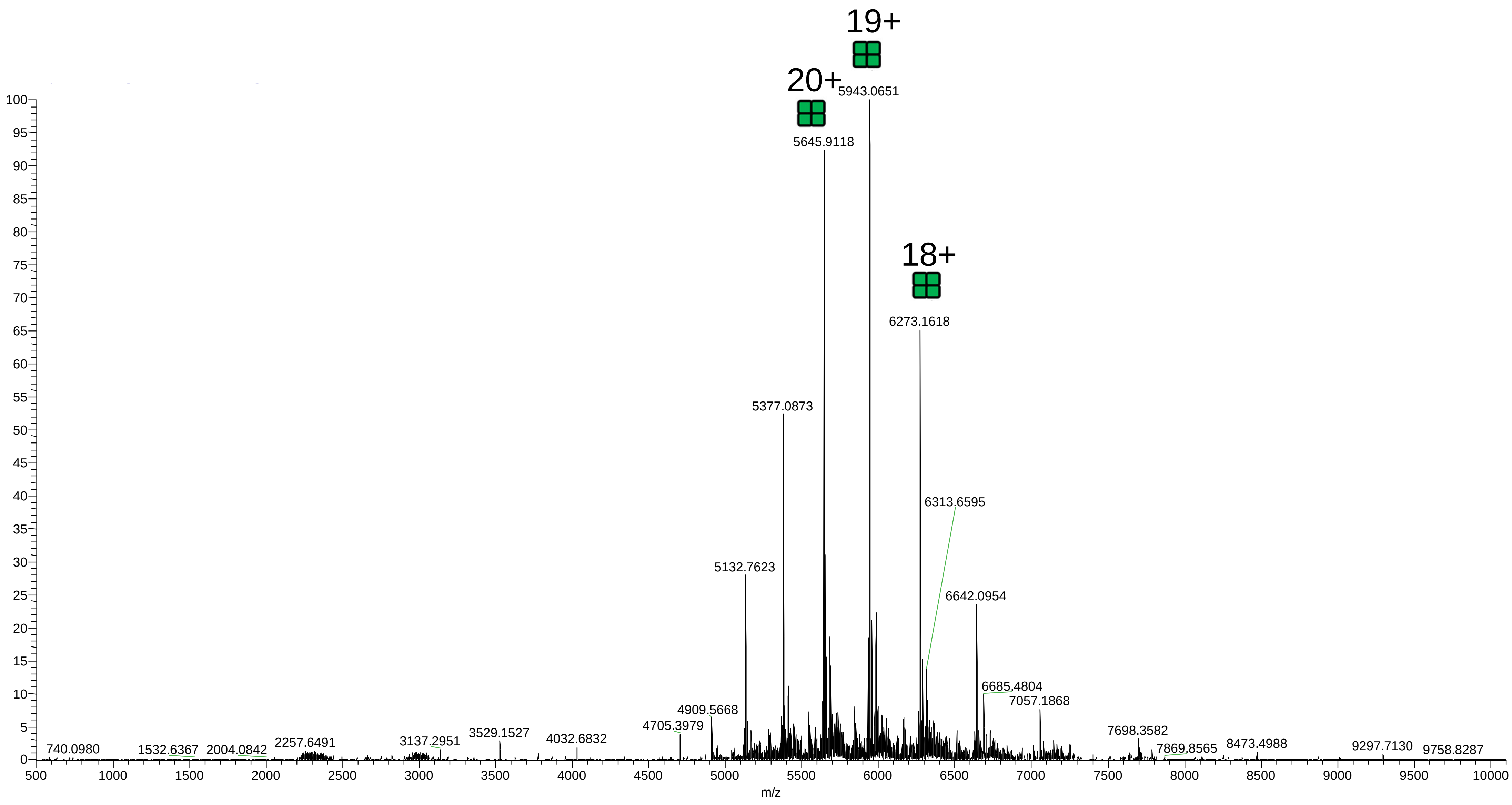

Supplementary Figure 29: Extended mass spectrum of purified tetrameric AQP0 incubated with cortical lens lipid extract.

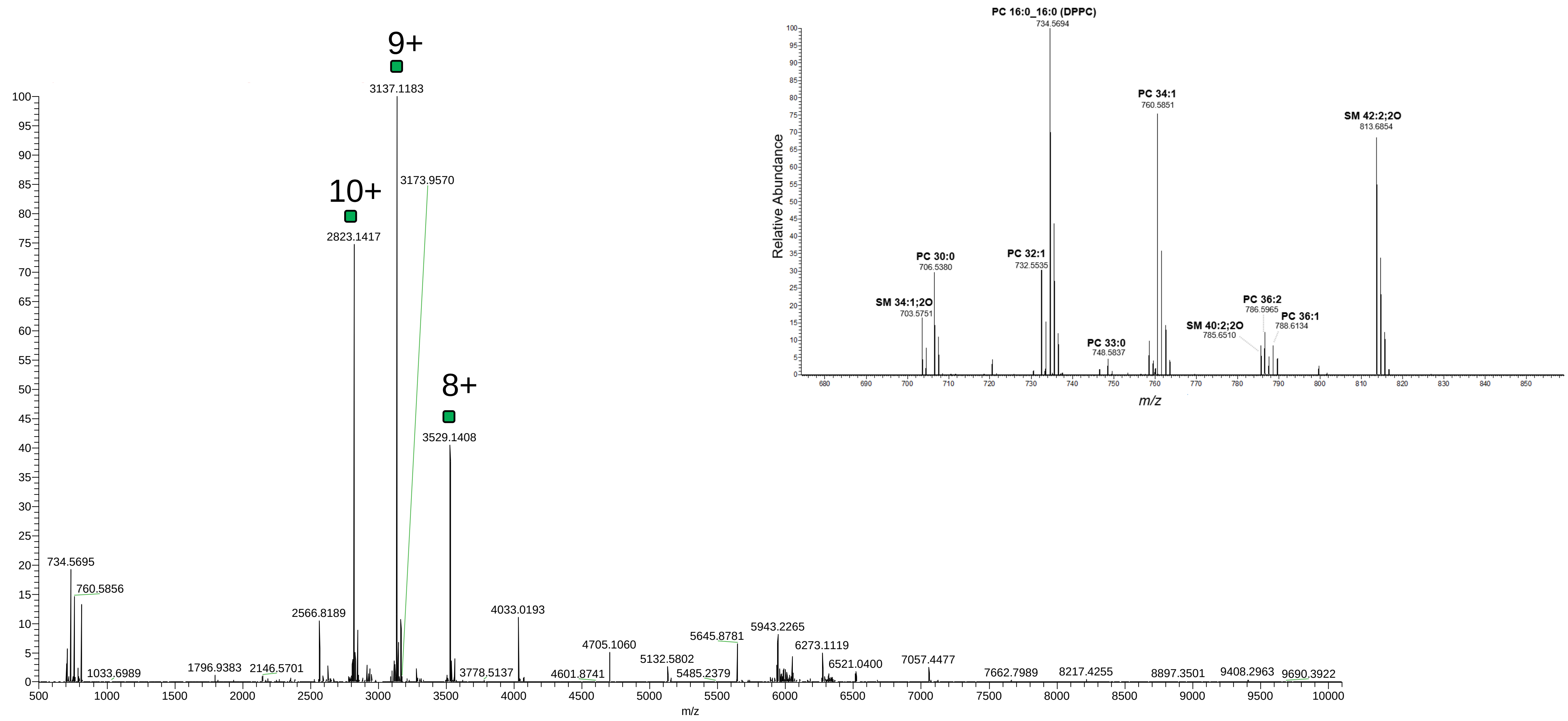

Supplementary Figure 30: Tandem mass of AQP0 incubated with cortical lens lipid extract after fragmentation at 200V of isolation window highlighted in Figure 4B.

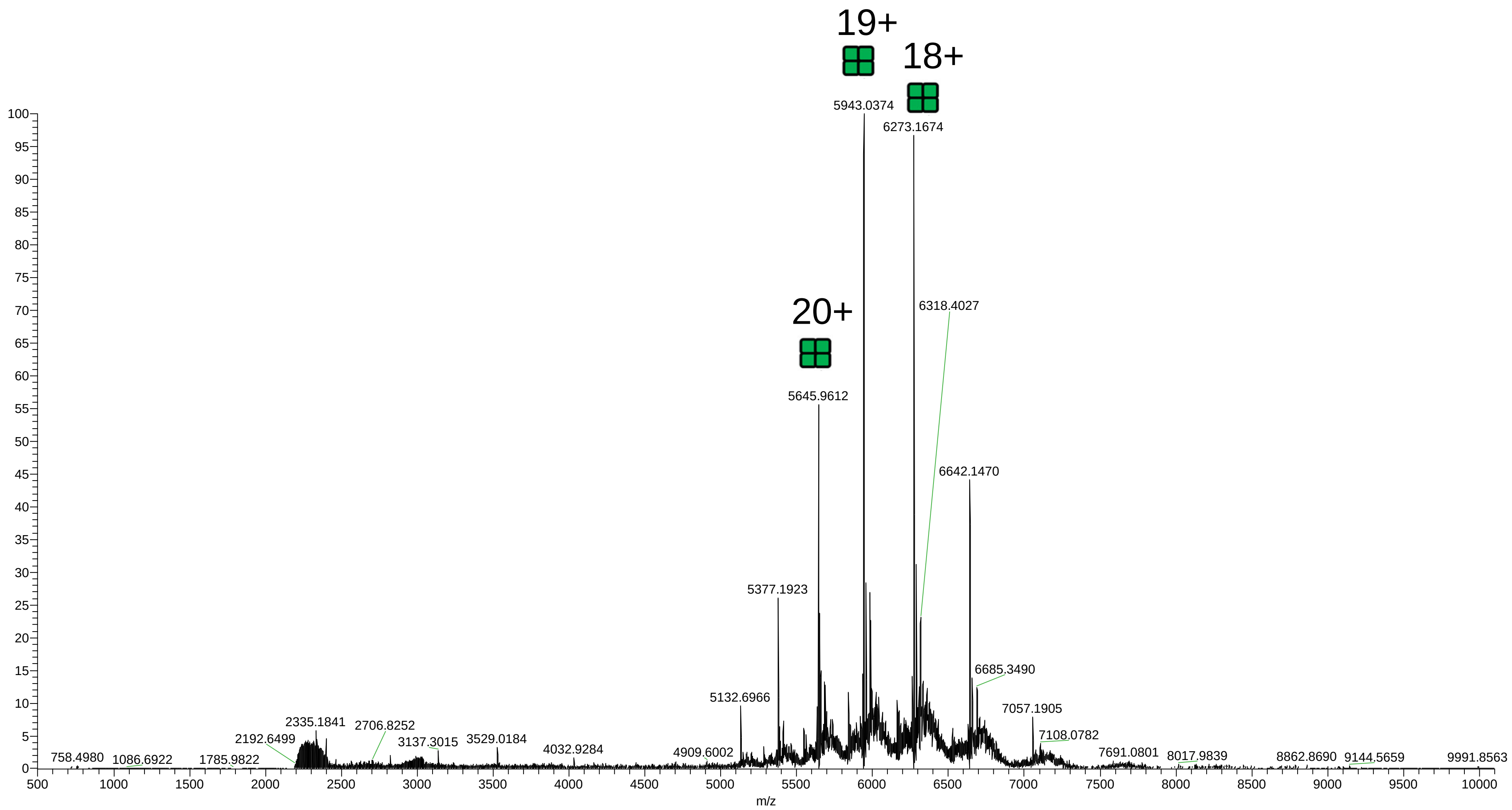

Supplementary Figure 31: Extended mass spectrum of purified AQP0 incubated with nucleus lens lipid extract.

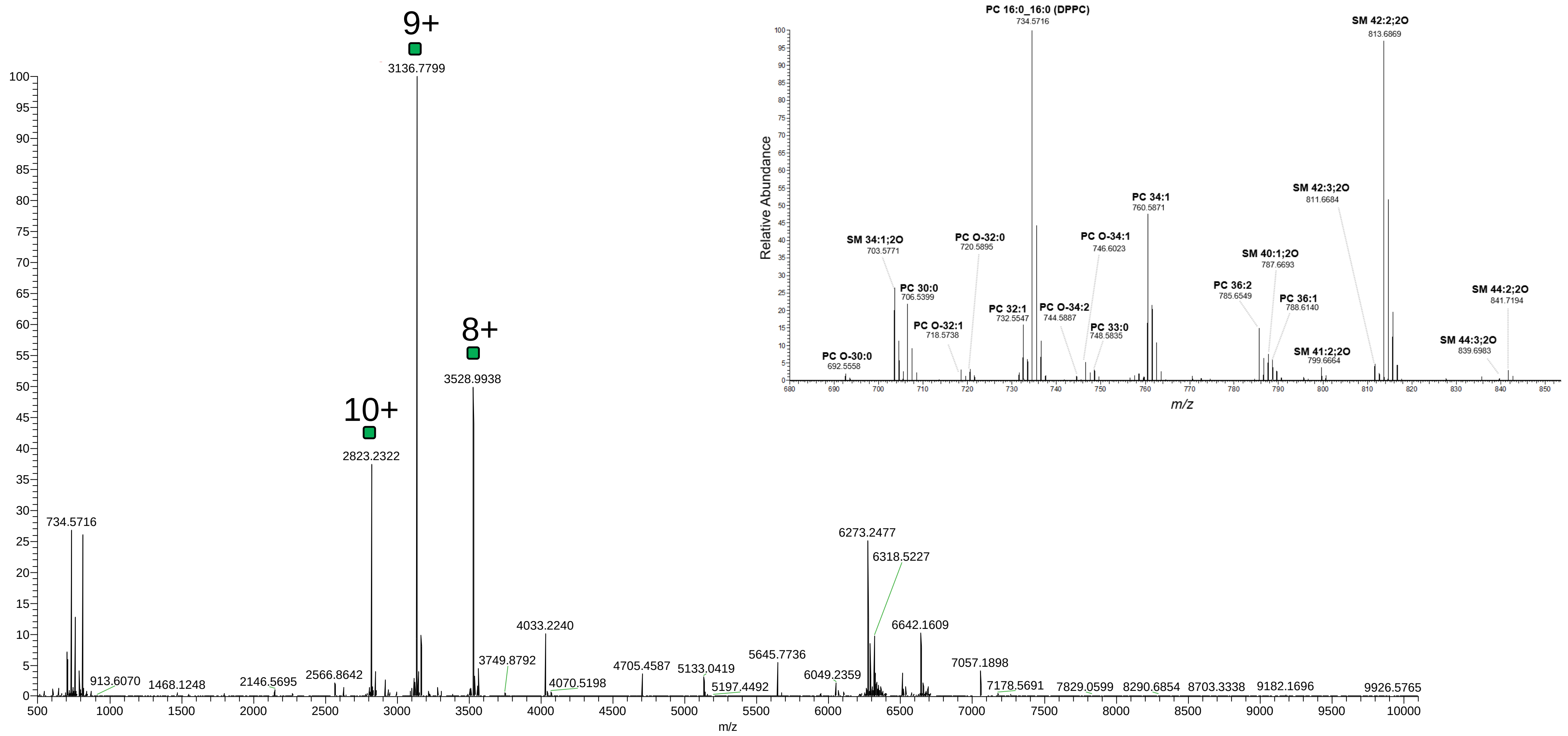

Supplementary Figure 32: Tandem mass spectrum of AQP0 incubated with nucleus lens lipid extract after fragmentation at 200V of isolation window highlighted in Figure 5B. Inset is a zoom-in of the low *m/z* range.

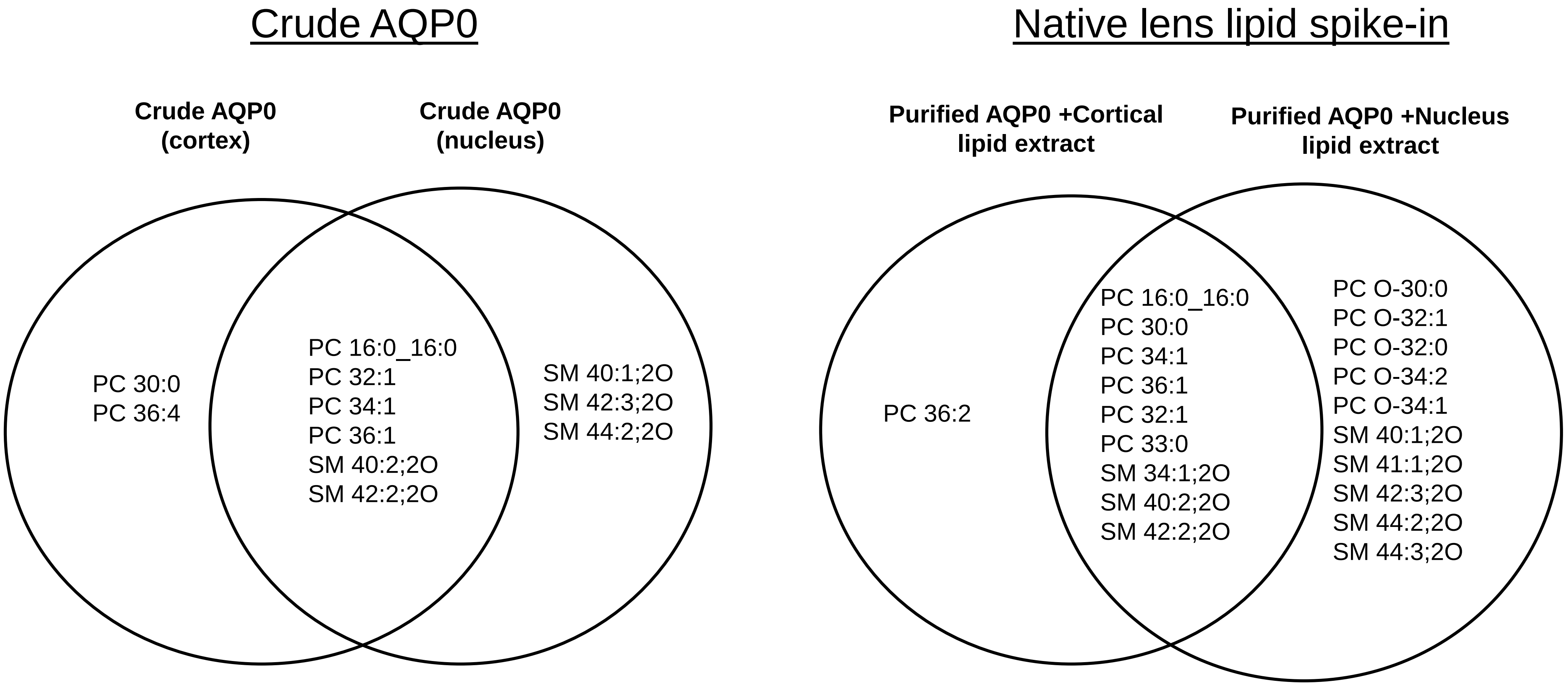

Supplementary Figure 33: Venn-diagram comparing AQP0-bound lipids in the crude cortex versus crude nucleus samples (left) and purified AQP0 incubated with a cortical versus nucleus lens lipid extract (right).

LC-MS/MS Lipid identification	Theoretical Monoisotopic *m/z* $[M+H]^+$	Experimental Monoisotopic *m/z* [M+H]	ppm
PC 30:0	706.5381	706.5406	3.5383
PC 32:1	732.5465	732.5503	5.1873
PC 16:0_16:0 (DPPC)	734.5694	734.5703	1.2252
PC 34:1	760.5851	760.5867	2.1036
PC 36:4	782.5694	782.5689	0.6389
SM 40:2;2O	785.6531	785.6557	3.3093
PC 36:1	788.6164	788.6419	1.9020
SM 42:2;2O	813.6844	813.6873	3.5640

Supplementary Table 1: Calculated mass error of LC-MS/MS identified lipids detected via MS of an AQP0 crude cortical bovine lens sample.

LC-MS/MS Lipid identification	Theoretical Monoisotopic *m/z* [M+H]+	Experimental Monoisotopic m/z [M+H]+	ppm
PC 32:1	732.5538	732.5570	4.3682
PC 32:0	734.5694	734.5728	4.6285
PC 34:1	760.5851	760.5859	1.0518
SM 40:2;2O	785.6531	785.6543	1.5273
SM 40:1;2O	787.6687	787.6672	1.9043
PC 36:1	788.6164	788.6189	3.1701
SM 42:3;2O	811.6687	811.6688	0.1232
SM 42:2;2O	813.6844	813.6906	7.6196
SM 44:2;2O	841.7157	841.7194	4.3957

Supplementary Table 2: Calculated mass error of LC-MS/MS identification of lipids detected via MS from an AQP0 crude nucleus lens sample.

LC-MS/MS Lipid identification	Theoretical Monoisotopic *m/z* $[M+H]^+$	Experimental Monoisotopic m/z $[M+H]^+$	ppm
SM 34:1;2O	703.5748	703.5751	0.4263
PC 30:0	706.5381	706.538	0.1415
PC 32:1	732.5538	732.5535	0.4095
PC 16:0_16:0 (DPPC)	734.5694	734.5695	0.1361
PC 33:0	748.5851	748.5837	1.8701
PC 34:1	760.5851	760.5856	0.6573
SM 40:2;2O	785.6531	785.651	2.6729
PC 36:2	786.6007	786.5965	5.3394
PC 36:1	788.6164	788.6134	3.8041
SM 42:2;2O	813.6844	813.6854	1.2289

Supplementary Table 3: Calculated mass error of LC-MS/MS identification of lipids detected via MS from a sample of purified AQP0 incubated with a cortical lens lipid extract.

LC-MS/MS Lipid identification	Theoretical Monoisotopic *m/z* [M+H]⁺	Experimental Monoisotopic *m/z* [M+H]+	ppm
PC O-30:0	692.5589	692.5558	4.4761
SM 34:1;2O	703.5748	703.5771	3.2690
PC 30:0	706.5381	706.5399	2.5476
PC O-32:1	718.5745	718.5738	0.9741
PC O-32:0	720.5902	720.5895	0.9714
PC 32:1	732.5538	732.5547	1.2285
PC 16:0_16:0 (DPPC)	734.5694	734.5716	2.9949
PC O-34:2	744.5902	744.5887	2.0145
PC O-34:1	746.6058	746.6023	4.6878
PC 33:0	748.5851	748.5835	2.1373
PC 34:1	760.5851	760.5871	2.6295
SM 40:2;2O	785.6531	785.6549	2.2910
SM 40:1;2O	787.6687	787.6693	0.7617
PC 36:1	788.6164	788.6140	3.0433
SM 41:2;2O	799.6687	799.6664	2.8761
SM 42:3;2O	811.6687	811.6684	0.3696
SM 42:2;2O	813.6844	813.6869	3.0724
SM 44:3;2O	839.7001	839.6983	2.1436
SM 44:2;2O	841.7157	841.7194	4.3957

Supplementary Table 4: Calculated mass error of LC-MS/MS identification of lipids detected via MS from a sample of purified AQP0 incubated with a nucleus lens lipid extract.
